# Carbamazepine Induces Focused T Cell Responses in Resolved Stevens-Johnson Syndrome and Toxic Epidermal Necrolysis Cases But Does Not Perturb the Immunopeptidome for T Cell Recognition

**DOI:** 10.3389/fimmu.2021.653710

**Published:** 2021-04-12

**Authors:** Nicole A. Mifsud, Patricia T. Illing, Jeffrey W. Lai, Heidi Fettke, Luca Hensen, Ziyi Huang, Jamie Rossjohn, Julian P. Vivian, Patrick Kwan, Anthony W. Purcell

**Affiliations:** ^1^ Infection and Immunity Program, Department of Biochemistry and Molecular Biology, Biomedicine Discovery Institute, Monash University, Clayton, VIC, Australia; ^2^ Department of Microbiology and Immunology, Peter Doherty Institute for Infection and Immunity, University of Melbourne, Parkville, VIC, Australia; ^3^ Australian Research Council Centre of Excellence for Advanced Molecular Imaging, Monash University, Clayton, VIC, Australia; ^4^ Institute of Infection and Immunity, Cardiff University School of Medicine, Cardiff, United Kingdom; ^5^ Department of Neuroscience, Central Clinical School, Alfred Hospital, Monash University, Melbourne, VIC, Australia; ^6^ Departments of Medicine and Neurology, Royal Melbourne Hospital, University of Melbourne, Parkville, VIC, Australia; ^7^ Department of Medicine and Therapeutics, Prince of Wales Hospital, Chinese University of Hong Kong, Hong Kong, Hong Kong

**Keywords:** T cells, SJS, Stevens-Johnson syndrome, carbamazepine, drug hypersensitivity, immunopeptidomics, T cell receptor

## Abstract

Antiseizure medications (ASMs) are frequently implicated in T cell-mediated drug hypersensitivity reactions and cause skin tropic pathologies that range in severity from mild rashes to life-threatening systemic syndromes. During the acute stages of the more severe manifestations of these reactions, drug responsive proinflammatory CD8^+^ T cells display classical features of Th1 cytokine production (*e.g.* IFNγ) and cytolysis (*e.g.* granzyme B, perforin). These T cells may be found locally at the site of pathology (*e.g.* blister cells/fluid), as well as systemically (*e.g.* blood, organs). What is less understood are the long-lived immunological effects of the memory T cell pool following T cell-mediated drug hypersensitivity reactions. In this study, we examine the ASM carbamazepine (CBZ) and the CBZ-reactive memory T cell pool in patients who have a history of either Stevens-Johnson syndrome (SJS) or toxic epidermal necrolysis (TEN) from 3-to-20 years following their initial adverse reaction. We show that *in vitro* drug restimulation of CBZ-reactive CD8^+^ T cells results in a proinflammatory profile and produces a mainly focused, yet private, T cell receptor (TCR) usage amongst human leukocyte antigen (HLA)-B*15:02-positive SJS or TEN patients. Additionally, we show that expression of these CBZ-reactive TCRs in a reporter cell line, lacking endogenous αβTCR, recapitulates the features of TCR activation reported for ASM-treated T cell lines/clones, providing a useful tool for further functional validations. Finally, we conduct a comprehensive evaluation of the HLA-B*15:02 immunopeptidome following ASM (or a metabolite) treatment of a HLA-B*15:02-positive B-lymphoblastoid cell line (C1R.B*15:02) and minor perturbation of the peptide repertoire. Collectively, this study shows that the CBZ-reactive T cells characterized require both the drug and HLA-B*15:02 for activation and that reactivation of memory T cells from blood results in a focused *private* TCR profile in patients with resolved disease.

## Introduction

Antiseizure medications (ASMs) are routinely used to treat epilepsy and other neuropsychiatric conditions, such as neuropathic pain and bipolar affective disorder. Commonly prescribed ASMs, including carbamazepine (CBZ) and phenytoin (PHT), have been implicated in life-threatening drug hypersensitivity reactions (DHRs) that predominantly target the skin (1). These drug reactions typically occur in the first 2-3 months of drug administration and span a range of clinical pathologies, including mild rash [*e.g.* maculopapular exanthema (MPE)], systemic symptoms [*e.g.* drug reaction with eosinophilia and systemic symptoms (DRESS)], as well as more severe bullous reactions involving rapid development of blisters and lesions accompanied by skin detachment [*e.g.* Stevens-Johnson syndrome (SJS) and toxic epidermal necrolysis (TEN)] (2–5). Whilst early drug withdrawal can ameliorate symptoms in milder cases, the more severe cutaneous reactions require specific clinical treatments and hospitalization for disease resolution.

ASMs can be segregated into two main groups, aromatic and non-aromatic compounds. Reports have shown that aromatic compounds [*e.g.* CBZ, oxcarbazepine (OXC; a structural derivative of CBZ), PHT, lamotrigine, phenobarbital] are highly associated with cutaneous DHRs (1) in individuals of particular ethnicities driven by their human leukocyte antigen (HLA) genetic profile. In contrast, the non-aromatic ASMs (*e.g.* valproic acid, gabapentin) are considerably less associated with DHRs [extensively reviewed in (6)]. The greatest risk association reported for CBZ-induced SJS/TEN is expression of HLA-B*15:02 in individuals of Han Chinese descent (Odds Ratio 895) (7), as well as other Asian ethnicities including Thai, Malaysian and Indian (8–10). Indeed, carriers of the broad HLA-B75 serotype (includes B*15:02, B*15:08, B*15:11, B*15:21) are also adversely affected by CBZ treatment (8, 10–14). HLA-A allotype risk associations for HLA-A*31:01, -A*24:02 and -B*57:01 have also been reported for CBZ-induced SJS/TEN, DRESS and MPE across different ethnicities (Han Chinese, Korean, European, Japanese) (13, 15–20), with lower risk associations.

Small molecule drugs, such as ASMs, can promote immune reactions *via* T cell activation. ASM-induced T cell activation is proposed to occur *via* non-covalent and labile interactions between the drug, or a metabolite of the parent drug, and the HLA/peptide complex and T cell receptor (TCR) (21). Additionally, whilst peptide occupancy of the HLA molecule is necessary, there is no requirement for *de novo* peptide/HLA complex formation (21) and the drug does not markedly alter the anchor residue preference of HLA-B*15:02 suggesting it is not binding within the primary anchor pockets during peptide loading (22). Contesting the latter, a recent report has suggested that the CBZ metabolite, carbamazepine-10,11-epoxide (ECBZ), is capable of altering the HLA-B*15:02 immunopeptidome and anchor residue prevalence (23). To date there is no published structure of the interaction of CBZ and the peptide/HLA and/or TCR, although diverse *in silico* models have been generated ranging from our previous proposal of occupation of the antigen-binding cleft, to more surface exposed positions, or, with the additional inclusion of identified drug-reactive TCRs, the HLA-TCR interface (21, 22, 24).

In contrast to the antiretroviral drug abacavir, which drastically alters the immunopeptidome and facilitates a diverse and polyclonal T cell response (25–27), examination of CBZ-induced T cells revealed a much more focused TCR usage associated with SJS/TEN (28). A recent study utilized next generation sequencing to examine the blister cells of CBZ-induced SJS/TEN patients identifying a public HLA-B*15:02-restricted αβTCR [complementarity determining region (CDR)3 sequence; TCRα VFDNTDKLI and TCRβ ASSLAGELF] that also recognized structural analogs of CBZ (24). These TCR signatures contrast with an early report demonstrating the expansion of VA-22 and VB-11-ISGSY dominant clonotypes derived from the blister fluid of SJS/TEN patients recruited in Taiwan expressing HLA-B*15:02 (28). These TCRs were identified using traditional Sanger sequencing and were confounded by *in vitro* T cell co-culture with antigen presenting cells (APCs), which has been suggested to bias the outgrown T cell repertoire (29). Another study examining the *ex vivo* TCRβ repertoire in peripheral blood mononuclear cells (PBMCs) isolated from CBZ-induced SJS (n=5) or TEN (n=1) patients showed that diversity was directly linked to disease severity, with the TEN patient having a significantly decreased TCRβ repertoire compared to SJS patients (30).

What is less understood are the long-lived immunological features of the CBZ-responsive memory T cell pool in patients with resolved disease. Do these patients exhibit similar immunological profiles to those reported during active disease, such as effector functions including Th1 cytokine production (*e.g.* IFNγ) (28) and cytolytic molecules (*e.g.* granzyme B, perforin) (31), TCR repertoire clonality, mode of drug recognition and cross-reactivity towards other ASMs? This study shows that the *in vitro* drug expanded TCR repertoire of resolved CBZ-induced SJS or TEN patients remains relatively clonal years after acute disease. These T cells respond to HLA-B15-positive APCs in the presence of CBZ, as well as related compounds, a finding that is recapitulated by expression of these cloned TCRs in a reporter cell line. Here, the responses required the continuous presence of soluble drug and HLA-B15 expression, without the need for *de novo* generation of the peptide/HLA complex. No marked drug-induced alteration in peptide anchor preference of HLA-B*15:02 was induced by any of CBZ, the metabolite ECBZ, or the structurally related ASM OXC. Together these data support the presence of a long-lived memory pool of CBZ responsive T cells in SJS or TEN patients, which are activated by structurally related ASMs in the absence of marked alteration of the immunopeptidome.

## Materials and Methods

### Study Cohort

Eight patients were recruited from Hong Kong, with six HLA-B*15:02-positive patients experiencing CBZ-induced SJS or TEN, one HLA-B*15:02-negative patient with CBZ-induced SJS and one CBZ-tolerant (**Table 1**, **Supplementary Table 1**). As described in our previous studies (32, 33) the diagnosis of SJS or TEN was based on the criteria by Roujeau and Stern (2) defined by skin detachment in two or more mucosal sites, and was confirmed by dermatologists. Healthy HLA-B*15:02^+^ individual controls (AP numbers; n=7; **Table 1**) were also recruited from both Monash University and the Australian Bone Marrow Donor Registry. All study participants provided written consent, with ethics approval granted by the Joint Chinese University of Hong Kong-New Territories East Cluster Clinical Research Ethics Committee (Hong Kong; CRE-2006.203 for patients), Monash University (Victoria, Australia; HREC-4717 for healthy individuals) and the Australian Bone Marrow Donor Registry (New South Wales, Australia; 2013/04 for healthy individuals).

**Table 1:** Study participants: cases and healthy donors.

Participant	ID	Gender	CBZ exposure	Reaction	HLA-B*15:02	Post-reaction sample collection (months)
Cases	T00016	Female	Yes	SJS	Positive	49
	T00024	Female	Yes	SJS	Positive	39
	E10056	Female	Yes	TEN	Positive	129
	E10076	Male	Yes	SJS	Positive	58
	E10314	Male	Yes	SJS	Negative	166
	E10367	Female	Yes	SJS	Positive	144
	E10630	Female	Yes	SJS	Positive	246
	E10493	Female	Yes	Tolerant	Negative	N/A
Healthy donors	AP005	Unknown	No	N/A	Positive	N/A
	AP017	Unknown	No	N/A	Positive	N/A
	AP022	Unknown	No	N/A	Positive	N/A
	AP026	Unknown	No	N/A	Positive	N/A
	AP027	Unknown	No	N/A	Positive	N/A
	AP029	Unknown	No	N/A	Positive	N/A
	AP102	Female	No	N/A	Positive	N/A

N/A, not applicable; SJS, Stevens-Johnson syndrome; TEN, toxic epidermal necrolysis.

### Cell Preparation and *In Vitro* Expansion of Drug-Induced T Cells

Blood samples were collected from study participants, with PBMCs isolated using Ficoll-Paque (GE Healthcare, Uppsala, Sweden) density gradient centrifugation and either used immediately or cryopreserved in fetal calf serum (FCS) containing 10% DMSO (Sigma-Aldrich, MO, USA) at -196°C until required. Before use for T cell stimulation, PBMCs were quickly thawed in 37°C and washed twice in RPMI 1640 (Gibco, Life Technologies, NY, USA) and resuspended in Complete Medium [RPMI 1640 supplemented with 2 mM MEM nonessential amino acid solution (Gibco), 100 mM HEPES (Gibco), 2 mM L-glutamine (Gibco), Penicillin/Streptomycin (Gibco), 50 μM 2-mercaptoethanol (Sigma-Aldrich) and 10% heat inactivated human blood group AB serum (Sigma-Aldrich)].

Drug-induced T cells were *in vitro* expanded following PBMC stimulation at a density of 5 million per 2 mL of Complete Medium in a 24-well plate with 25 μg/mL of either CBZ (Sigma) or the metabolite ECBZ (Sigma or SYNthesis med chem, Australia). On days 4 to 14, T cell cultures were supplemented with 50 U/mL of recombinant human IL-2 (Peprotech, NJ, USA) and subcultured as required to ensure optimal outgrowth.

### Antigen Presenting Cells and HLA Expression

C1R.B*15:01, C1R.B*15:02, C1R.B*15:21, C1R.B*15:25 and C1R.B*08:01 transfectants and transductants were generated from the HLA class I-reduced C1R B-lymphoblastoid cell line, which has minimal HLA-B*35:03 and normal levels of HLA-C*04:01 cell surface expression (34, 35). All APCs were cultured in RF10 [same constituents as Complete Medium except 10% heat inactivated FCS (Sigma-Aldrich)]. Maintenance of transfected HLA expression (except green fluorescent protein (GFP) tagged C1R.B*15:21 and C1R.B*15:25) during long-term culture was facilitated by selection antibiotics [Geneticin G418 (0.5 mg/ml; Roche Diagnostics, Mannheim, Germany) or hygromycin B (0.3 mg/ml; Life Technologies, Carlsbad, CA)] as required. GFP expression is used as a reporter of HLA expression facilitating flow cytometric sorting. Increased HLA-I expression (compared to C1R Parental) was confirmed *via* flow cytometry by indirect staining with appropriate antibodies; anti-human pan HLA-I (W6/32 hybridoma; **Supplementary Figure 1A**), anti-human HLA-Bw6 (HB152 hybridoma; **Supplementary Figure 1B**) and a secondary goat anti-mouse IgG phycoerythrin (PE) (1:200 dilution; Southern Biotech, Birmingham, AL). All hybridomas were produced in-house. Stained cells were acquired on LSRII flow cytometer [Becton Dickinson (BD), San Jose, CA]. Flow cytometry data was analyzed using FlowJo software (version 10, BD).

### Drug-Pulsed APCs and T Cell Stimulation

Functionality of the drug-induced T cells, including cross-reactivity, was assessed using intracellular cytokine staining (ICS) for production of either IFNγ and/or TNF (36). Briefly, day 14 T cells (2x10^5^) were restimulated with Dynabeads^®^ Human T-Activator CD3/CD28 beads (positive control; Life Technologies), drug alone (25 μg/mL), APC alone (1x10^5^; APC) or APC in the presence of drug (1x10^5^; APC+25 μg/mL drug) for a total of six hours. Brefeldin A (10 µg/mL; Sigma-Aldrich) was added for the last 4 hours of co-culture. Cells were then surface labeled with LIVE/DEAD^®^ fixable Aqua stain (Life Technologies), CD4 PE (clone RPA-T4), CD8 PerCP-Cy5.5 (clone SK1), fixed in 1% paraformaldehyde (ProSciTech, Queensland, Australia) in phosphate buffered saline (PBS) and then permeabilized with 0.3% Saponin (Sigma-Aldrich) containing IFNγ PE-Cy7 (clone B27) and TNFα V450 (clone MAb11) before acquisition on a LSRII flow cytometer [Becton Dickinson (BD), San Jose, CA, USA] (**Supplementary Figure 2A**). All monoclonal antibodies were purchased from BD and titrated for optimal staining efficiency. A maximum of 50,000 lymphocytes were acquired on a BD LSRII flow cytometer utilizing BD FACSDIVA™ software (FlowCore, Monash University) and analyzed using FlowJo software (version 10, BD). Representative gating strategy is shown in **Supplementary Figure 2B**.

### Paired αβTCR Analysis of Drug-Induced T Cells

A single-cell sort was performed to characterize the αβTCR signature of drug-induced T cells using the IFNγ Secretion Assay–Detection Kit (allophycocyanin; Miltenyi Biotec, CA, USA). Cryopreserved T cell lines were thawed and rested overnight in Complete Medium. T cell lines (maximum of 5x10^6^ cells) were incubated with either C1R.B*15:02 alone or C1R.B*15:02 + drug (25 μg/mL) target cells (2:1 ratio) in RH5 media (same constituents as Complete Medium, except 5% heat inactivated human blood group AB serum) for 4 hours at 37°C, 5% CO_2_. Cells were washed in cold Wash Buffer (0.5% FCS, 2 mM EDTA pH 8.0 in PBS), centrifuged (285 ×*g*, 5 min, 4°C) and supernatant aspirated before addition of IFNγ catch reagent antibody according to manufacturer’s instructions. Cells were incubated on ice for 5 min and topped up to 10 mL with warm RH5 media, and drug (25 μg/mL) was re-added. Cells were incubated for 45 min at 37°C, 5% CO_2_ with rotation. Cells were washed in cold Wash Buffer, centrifuged and supernatant aspirated prior to co-staining with IFNγ allophycocyanin detection reagent and CD8 FITC (clone HIT8a). Cells were incubated on ice for 20 min, washed in cold Wash Buffer, centrifuged and resuspended in 300 μL cold Wash Buffer (**Supplementary Figure 2C**).

Single cells were sorted on a BD Influx flow cytometer (FlowCore, Monash University) directly into 96-well PCR plates (Bio-Rad, Hercules, CA, USA) based on CD8^+^IFNγ^-^ for drug-unresponsive T cells (negative control) and CD8^+^IFNγ^+^ for drug-induced T cells (**Supplementary Figure 2C**). Sorted plates were immediately stored at -80°C until required. Analysis of paired TCRα and β genes was carried out by multiplex nested RT-PCR and sequencing of α and β products as described previously (37). Both external and internal rounds of PCR included 40 TRAV and 27 TRBV forward primers, and a TRAC and TRBC reverse primer, as detailed elsewhere (37). Sequences were analyzed according to the ImMunoGeneTics/V-QUEry and STandardization web-based tool (38). All TCR nomenclature was according to Folch et al. (39). CDR3 amino acid sequences described within the text start from CDR3-position 3, which is equivalent to amino acid position 107 of the TRAV and TRBV segments, and end at TRAJ-position 10 or TRBJ-position 6.

### Generation of SKW3.TCR Cell Lines

Full-length human TCRα and TCRβ cDNA was cloned into pMIG vector separated by a self-cleaving 2A peptide as described previously (40, 41). A pMIG vector, with IRES-linked GFP expression, containing a specific TCR for AP026/CBZ, E10056/CBZ, or E10630/CBZ (4 μg) was retrovirally transduced into SKW3.hCD8αβ cells (kindly provided by Dr. Zhenjun Chen, Peter Doherty Institute for Infection and Immunity, University of Melbourne; hereafter referred to as SKW3), which is negative for endogenous TCRαβ but contains CD3 and its signaling components, using HEK293T packaging cells, pEQ-pam3(-E) (4 μg) and pVSV-G (2 μg) packaging vectors and Lipofectamine 3000 as previously described (41). The original SKW3 parental cell line was kindly provided by Dr. Klaus Steube, Leibniz Institute DSMZ-German Collection of Microorganisms and Cell Cultures (Braunschweig, Germany). The HLA typing of SKW3 cells is HLA-A*11:01, 30:01; B*35:01, 44:02; C*04:01, 05:01; DRB1*01:03, 04:01; DQB1*03:01; DPB1*04:01, 04:02. A non-specific control, SKW3.LC13 cell line, specific for an EBV epitope FLRGRAYGL (EBNA3A_325-333_) restricted to HLA-B*08:01 was generated in our previous study (41). The SKW3.TCR cell lines were maintained in RF10 media (**Supplementary Figure 1C**).

### Functional T Cell Assays

Activation of SKW3.TCR cells (1x10^5^) was assessed using cell-surface CD69 upregulation after 17-20 hours incubation with either C1R Parental, C1R.B*15:01, C1R.B*15:02, C1R.B*15:21, C1R.B*15:25 or C1R.B*08:01 targets (1:1 ratio) under different sets of conditions, including either direct drug addition (25 μg/mL) or by drug-pulsing APC (25 μg/mL) overnight then thorough washing in RPMI before SKW3.TCR co-incubation. SKW3.TCR cells were co-stained with CD3 PE-Cy7 (clone SK7), CD8 PerCP-Cy5.5 (clone SK1), CD69 APC (clone L78), LIVE/DEAD^®^ fixable Aqua stain. For all experiments, stimulation with Dynabeads^®^ Human T-Activator CD3/CD28 beads (Life Technologies) served as a positive control, and SKW3.TCR cells alone as a negative control. Flow cytometry data were acquired and analyzed as described previously (41). The CD69 expression profiles were measured as geometric mean fluorescence intensity (MFI) to provide more meaningful evaluation of changes in the relative amounts of expressed protein per cell. A maximum of 50,000 lymphocytes were acquired on a BD LSRII flow cytometer utilizing BD FACSDIVA™ software (FlowCore, Monash University) and analyzed using FlowJo software (version 10, BD).

### Immunopeptidome Analysis

The C1R.B*15:02 cell line was *in vitro* expanded in RF10, and treatment of cells with either CBZ (Sigma), ECBZ (Sigma or SYNthesis med chem, Australia) or OXC (SYNthesis med chem) for peptide elutions was performed at 25 μg/mL for 48 hours in roller bottles prior to harvesting. Cells (0.9-1.1 x 10^9^) were pelleted, washed twice in PBS and snap-frozen in liquid nitrogen. Cells were lysed, and the HLA class I isolated by immunoaffinity purification using solid-phase bound pan HLA class I antibody W6/32 as described previously (42). Peptides were dissociated using 10% acetic acid and separated from the HLA heavy and light (beta-2 microglobulin; β_2_m) chains by Reversed Phase HPLC (RP-HPLC) as described (43), monitoring elution by 215nm absorbance and collecting 500 μL fractions. The retention times of CBZ, ECBZ and OXC were determined through subjecting each molecule to the same RP-HPLC protocol. Peptide containing fractions, avoiding regions containing β_2_m and heavy chain, were vacuum concentrated and concatenated to generate 13 pools, including 3 pools aligned with the retention times of CBZ (pool 10), ECBZ (pool 12) and OXC (pool 13). Pools were vacuum concentrated to remove residual acetonitrile (ACN) and reconstituted in 15 μL 2% ACN, 0.1% formic acid (FA), spiked with 250 fmol iRT peptides (44).

Pools were analyzed by liquid chromatography-tandem mass spectrometry (LC-MS/MS) using a data dependent acquisition (DDA) strategy on a Q-Exactive Plus Hybrid Quadrupole Orbitrap (Thermo Fisher Scientific) utilizing a Dionex UltiMate 3000 RSLCnano system (Thermo Fisher Scientific). 5-6 µL of concentrated material was loaded onto an Acclaim PepMap 100 (100 µm x 2 cm, nanoViper, C18, 5 µm, 100Å; Thermo Scientific) in 2% ACN, 0.1% FA at a flow rate of 15 µL/min. Peptides were separated over an Acclaim PepMap RSLC (75 µm x 50 cm, nanoViper, C18, 2 µm, 100Å; Thermo Scientific) at 250 nL/min using a gradient of Buffer A (0.1% FA) and Buffer B (80% ACN, 0.1% FA) as follows: 2.5-7.5% B in 1 min, 7.5-32.5% B in 55 min, 32.5-40% B in 5 min, 40-99% B in 5 min, 99% B for 6 minutes, and 99-2.5% B in 1 min, prior to re-equilibration at 2.5% B for 20 min. Data were collected in positive mode: MS1 resolution 70,000, scan range 375-2000 m/z; MS2 resolution 17500, scan range 200-2000 m/z. The top 12 ions of +2 to +5 charge per cycle were chosen for MS/MS with a dynamic exclusion of 15 seconds.

Peptide sequences were assigned using PEAKS X+ (Bioinformatics Solutions Inc.) *via* a database search against the reviewed human proteome (UniProt/Swiss-Prot, accessed October 2018), and a database of common contaminants. The following settings were employed: Instrument – OrbiTrap (Orbi-Orbi), Fragment – HCD, Acquisition - DDA, Parent Mass Error Tolerance - 20.0 ppm, Fragment Mass Error Tolerance - 0.02 Da, Precursor Mass Search Type - monoisotopic, Enzyme - None, Variable Modifications - Oxidation (M) 15.99, Deamidation (NQ) 0.98, and Cysteinylation: 119.00, Max Variable PTM Per Peptide - 3. Peptides assigned at a 5% peptide false discovery rate (FDR) were utilized in downstream analyses. DDA data from previous analyses of the immunopeptidome of endogenous HLA I and II of C1R cells (43) were searched separately *via* the same pipeline as control datasets.

To characterize the HLA-B*15:02 binding motif, peptide sequences identified at a 5% peptide FDR in control data sets representing the endogenous HLA class I and II of C1R cells were removed. So too were peptides assigned without a protein accession or mapping exclusively to proteins in the contaminant database. For peptide counts, motif and length distribution analysis, only 7-20mer peptides were considered and peptides sequences containing deamidations or cysteinylations were treated as distinct from the native sequence. Figures were generated using Prism 9.0, GraphPad software (San Diego, CA). The mass spectrometry proteomics data have been deposited to the ProteomeXchange Consortium *via* the PRIDE (45) partner repository with the dataset identifier PXD023545 and 10.6019/PXD023545.

### Identification of Co-Purified Drugs/Metabolites

Drugs/metabolites were detected using a targeted LC-multiple reaction monitoring-MS (LC-MRM-MS) approach on a SCIEX QTRAP® 6500 plus mass spectrometer, coupled to an Ekspert™ nanoLC 415. For pools aligned with the RP-HPLC retention time of CBZ (pool 10), ECBZ (pool 12) and OXC (pool 13), residual sample post-peptide analysis was diluted by a factor of ~2. 6 μL diluted sample was loaded onto a NanoLC Trap ChromXP C18 column (350 μm x 0.5 mm, 3 µm particle size, 120 Å pore size) in 2% ACN, 0.1% FA at a flow rate of 5 µL/min for 5 minutes, prior to separation over a ChromXP nanoLC C18 column (75 µm x 15 cm, 3 µm particle size, 120 Å pore size) at 300 nL/min using an increasing gradient of Buffer B (80% ACN, 0.1% FA): 0–1 min 2% B, 1-2 min 2–12% B, 2–30 min 12–35% B, 30–50 min 35–80% B, 50–54 min hold at 80% B, 54-55 min 80-2% B, 55-65 min re-equilibration at 2% B. The QTRAP® 6500 was operated in positive mode, MRM scan type, in unit resolution for Q1 and Q3. For detection of CBZ three transitions were set with a Q1 mass of 237.102, and Q3 masses of 192.082 [Collision Energy (CE) 30], 194.097 (CE 25) and 220.077 (CE 15). For ECBZ and OXC, five transitions were set with a Q1 mass of 253.097, and Q3 masses of 180.082 (CE 30), 182.096 (CE 25), 208.076 (CE 30), 210.092 (CE 25) and 236.071 (CE 25). CE values were optimized based on injection of solubilized drug/metabolite. Three transitions for each iRT peptide were also monitored. Drug peaks areas are the sum peak areas of the monitored transitions. Drug peak areas were normalized to the sum peak areas of iRT peptides B-G (GAGSSEPVTGLDAK, VEATFGVDESNAK, YILAGVENSK, TPVISGGPYEYR, TPVITGAPYEYR, DGLDAASYYAPVR). Figures were generated using Prism 9.0, GraphPad software.

### Statistical Analysis

All data were reported as mean ± standard error of the mean (SEM), unless stated otherwise. Statistical significance was determined by nonparametric one-way ANOVA with post-hoc Tukey’s multiple comparison test, Mann-Whitney test or multiple comparisons using Holm-Sidak method (Prism 8.0, GraphPad software) with *p<0.05, **p<0.005 and ****p<0.0001. Standard error of the difference between mean amino acid prevalence was calculated using the Welch t-test (Prism 9.0, GraphPad software).

## Results

### Drug-Induced Recall Responses Are Restricted to CD8^+^ T Cells in Recovered SJS or TEN Patients

**Figure 1:**
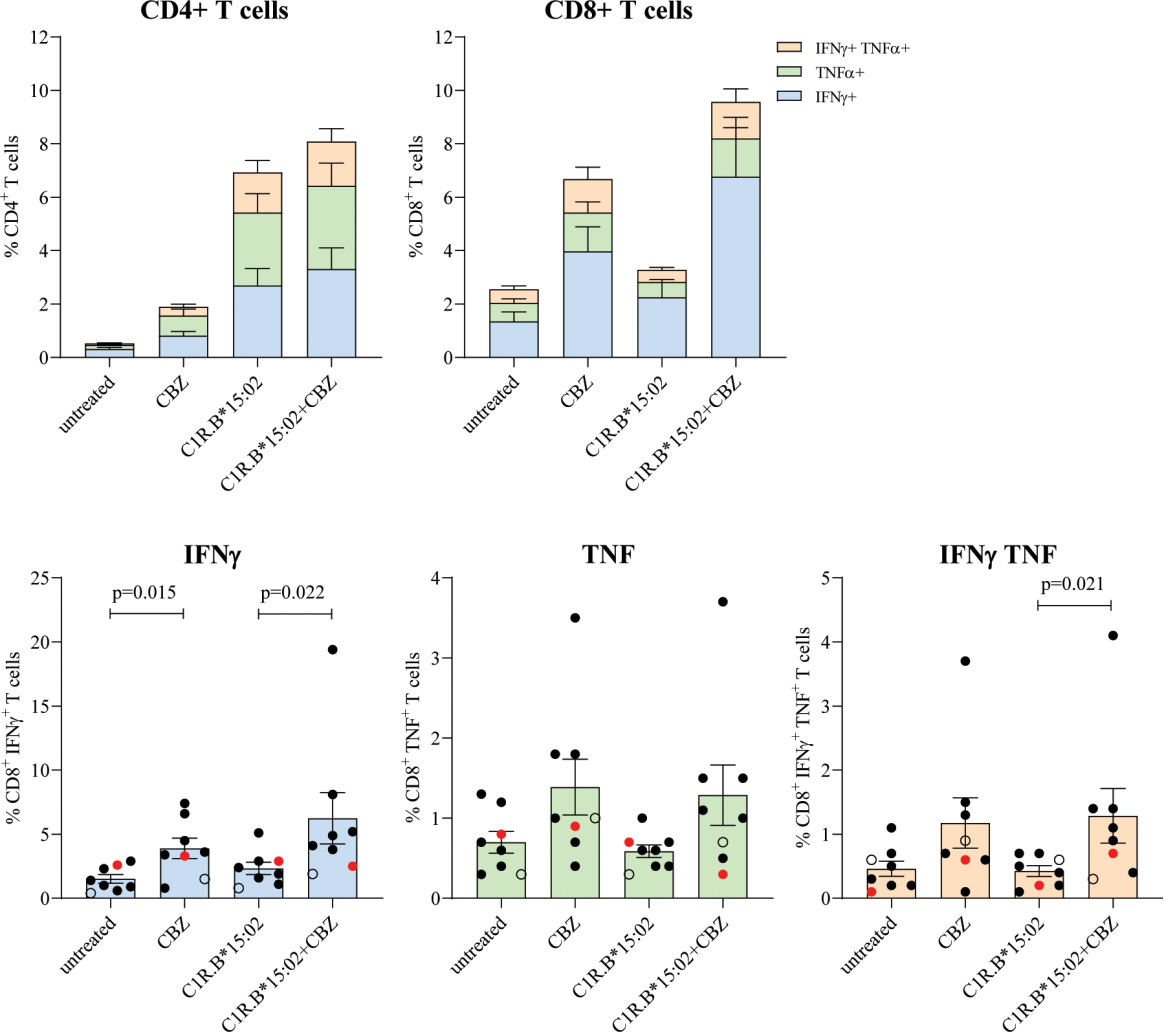
Restimulation of drug-reactive memory cells in recovered CBZ-induced SJS and TEN patients is restricted to CD8^+^ T cells. PBMCs were co-cultured in the presence of 25 μg/mL CBZ for 14 days. T cell subset phenotype (CD4^+^ or CD8^+^) and activation status (production of either IFNγ and/or TNFα) were measured following a 6-hour restimulation with either no drug (untreated), CBZ (25 μg/mL), C1R.B*15:02 or C1R.B*15:02+CBZ (25 μg/mL). Data was acquired on LSRII flow cytometer (BD) and analyzed using FlowJo software (version 10, BD). All data is expressed as mean ± SEM. **(A)** Grouped data demonstrated a drug-reactive response in CD8^+^ T cells expressing the different combinations of proinflammatory cytokines, but this was not observed for CD4^+^ T cells (n=7, single data). **(B)** Individual data (B*15:02 positive SJS or TEN ([n=6, solid black dots] and Tolerant [n=1, red dot], B*15:02 negative SJS [n=1, open black dot], single data) showed that activated CD8^+^ T cell produced significantly more IFNγ than TNFα (p-value <0.05; Mann-Whitney test; **B**).

We examined the immune reactivity profiles of convalescent SJS or TEN patients (7 cases and one drug-tolerant case) to determine whether they maintain a memory pool of T cells specific to the culprit drug that can be reactivated following subsequent drug exposures (**Table 1**). PBMCs treated with 25 μg/mL CBZ were expanded *in vitro* for 14 days. Outgrown CBZ-induced T cell lines were then restimulated in a 6-hour ICS assay with either no drug (untreated), CBZ (25 μg/mL), or with HLA-B*15:02^+^ APCs C1R.B*15:02 or C1R.B*15:02+CBZ (25 μg/mL) to measure activation *via* production of Th1 cytokines (IFNγ and TNF). Group data of the recovered patients showed that the CBZ-reactive T cell response, where activation was observed following CBZ and APC+CBZ stimulation, was primarily restricted to CD8^+^ T cells. However, the CD4^+^ T cells did show immune recognition of CBZ alone, likely *via* the mechanism of self-T cell presentation (*i.e.* T-T presentation). Here, 6 out of 7 patients expressing HLA-B*15:02 demonstrated non-specific CD4^+^ T cell responses towards the APCs most likely due to HLA class II mismatches expressed by the C1R.B*15:02 transfectant (**Figure 1A**). Optimization experiments, using healthy donors, showed similar CD4^+^ T cell immune responses (*i.e.* production of Th1 cytokines IFNγ and TNFα) to both the C1R parental and C1R.B*15:02 APC in either the presence or absence of CBZ (data not shown). Dissection of the individual CD8^+^ T cell responses profiled either by single or dual expression of IFNγ and TNF demonstrated significantly higher responses were driven by IFNγ production (p-value <0.05; Mann-Whitney test; **Figure 1B**). As expected, the drug-tolerant case did not respond to CBZ (red dot; **Figure 1B**).

### Priming of Drug-Naïve HLA-B*15:02^+^ Individuals Required Multiple Rounds of Drug Exposure

**Figure 2:**
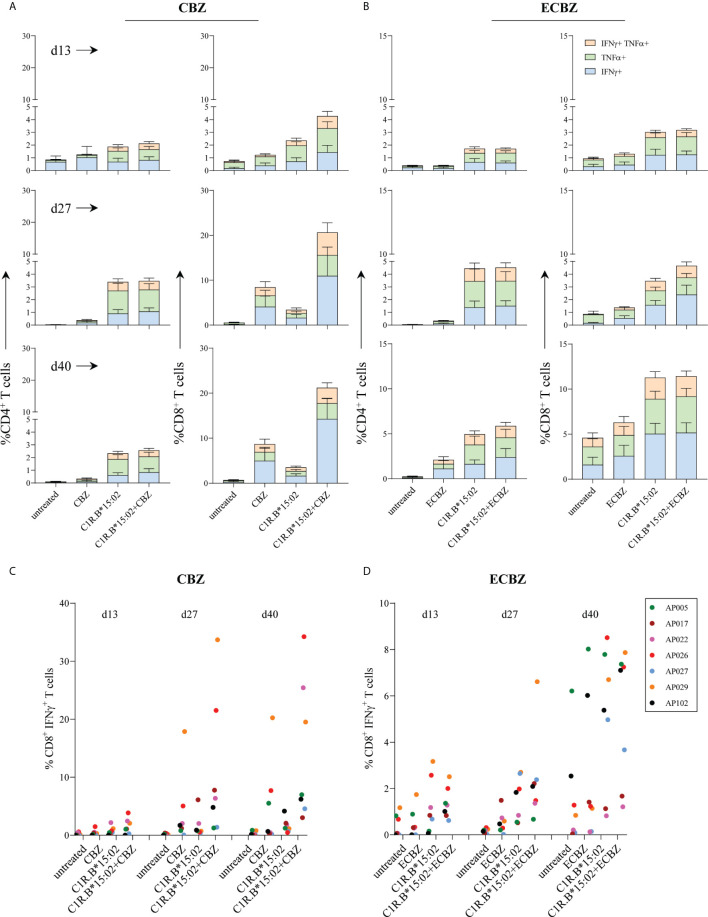
Time course of *in vitro* priming of drug-naïve HLA-B*15:02+ individuals. PBMCs derived from drug-naïve individuals were co-cultured in the presence of either 25 μg/mL CBZ or ECBZ for 13 to 40 days, with drug restimulation on days 13 and 27. T cell subset phenotype (CD4^+^ or CD8^+^) and activation status (production of either IFNγ and/or TNFα) were measured following a 6-hour restimulation with either no drug (untreated), drug (25 μg/mL; CBZ or ECBZ), C1R.B*15:02 or C1R.B*15:02+drug (25 μg/mL; CBZ or ECBZ). Data was acquired on LSRII flow cytometer (BD) and analyzed using FlowJo software (version 10, BD). All data is expressed as mean ± SEM. Grouped data demonstrated drug-reactive responses were restricted to CD8^+^ T cells expressing the different combinations of proinflammatory cytokines when primed with either **(A)** CBZ or **(B)** ECBZ (n=7, single data). Individual data tracking of immune reactivity to drugs demonstrated that multiple rounds of exposure with either **(C)** CBZ or **(D)** ECBZ were required for activation of CD8^+^ IFNγ^+^ T cells.

To demonstrate both the kinetics and magnitude of T cell activation following drug exposure we *in vitro* stimulated HLA-B*15:02^+^ drug-naïve PBMCs (n=7; **Table 1**) with either CBZ or its metabolite ECBZ at 25 μg/mL. On days 13 and 27, outgrown T cells were restimulated with drug (as per day 0) for continued *in vitro* expansion. Drug-induced T cell lines were tested prior to each restimulation and at day 40 (3 intervals total: day 13, 27 and 40) in a 6-hour ICS assay with either no drug (untreated), drug (CBZ or ECBZ; 25 μg/mL), or using C1R.B*15:02 or C1R.B*15:02+drug (CBZ or ECBZ; 25 μg/mL) as APC with activation quantitated by either IFNγ and/or TNF production. For CBZ-induced T cells, we observed similar findings to resolved cases (**Figure 1**) but with delayed kinetics as drug-specificity predominantly mediated by CD8^+^ T cells that was more pronounced by day 40 (**Figure 2A**). For ECBZ-induced T cells, the greatest magnitude of immune recognition was 2.7-fold lower than the CBZ parent drug on day 40 for IFNγ producing cells (C1R.B*15:02+drug; mean ± SEM: 14.28% ± 4.6 for CBZ and 5.16% ± 1.1 for ECBZ; p=0.0029 multiple comparisons using Holm-Sidak method) (**Figure 2B**). Although not all healthy donors demonstrated ECBZ-reactive CD8^+^ T cell responses. No drug-induced immunogenicity was shown by CD4^+^ T cells, with T-T presentation of CBZ or ECBZ resulting in minor responses (**Figures 2A, B**). Examination of individual data for CBZ-induced CD8^+^ IFNγ^+^ T cells showed strong responsiveness for both AP026 (21.5%) and AP029 (33.7%) on day 27 that equated to a 41.4-fold and 48.1-fold increase, respectively, over the APC background control (*i.e.* C1R.B*15:02 vs C1R.B*15:02+CBZ). This net effect was also demonstrated on day 40 for AP022 (25.4%), AP026 (34.2%) and AP029 (19.5%) with 14.1-fold, 72.9-fold and 17.3-fold increases, respectively (**Figure 2C**). Interestingly, the same level of *in vitro* expansion was not observed with the ECBZ metabolite, with only moderate CD8^+^IFNγ^+^ T cell responses measured (5-10% of total CD8^+^ T cells) on days 27 and 40, with high APC control background on day 40 being observed across all individuals (**Figure 2D**).

### CBZ-Reactive T Cell Cross-Reactivity Towards Tricyclic Aromatics

**Figure 3:**
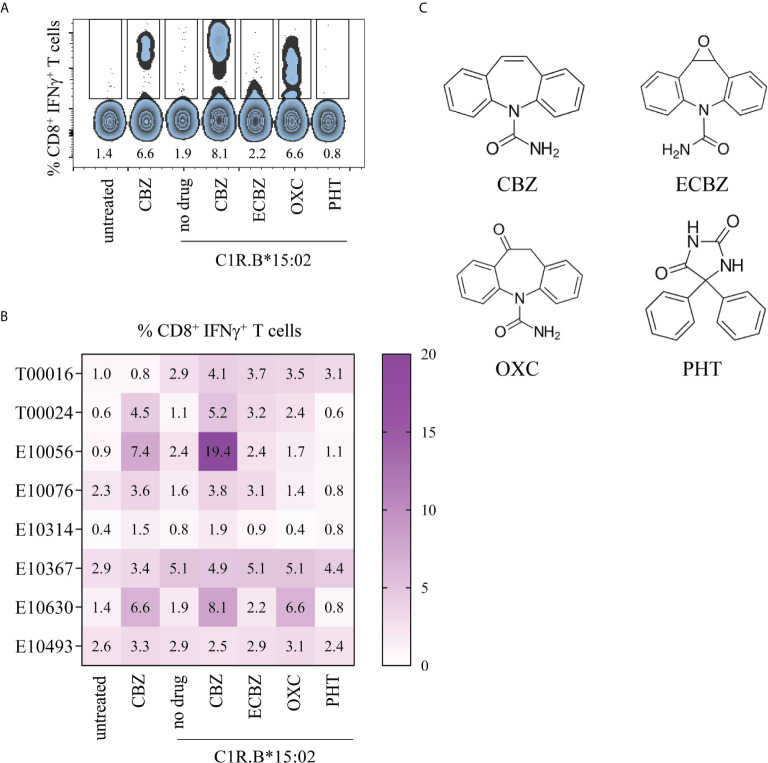
CBZ-reactive T cell cross-reactivity towards structures comprising a tricyclic ring. Day 14 CBZ-induced T cell lines derived from SJS and TEN patients (from **Figure 1**) were restimulated in a 6-hour ICS assay with media alone (untreated), CBZ (25 μg/mL) or C1R.B*15:02 APCs in the absence or presence of drug (25 μg/mL; CBZ, ECBZ, OXC, PHT) to measure CD8^+^ T cell activation *via* IFNγ production. Data was acquired on LSRII flow cytometer (BD) and analyzed using FlowJo software (version 10, BD). **(A)** Representative flow cytometric data for SJS patient E10630. **(B)** Matrix of CBZ-reactive T cell cross-reactivity towards ASMs with either bicyclic or tricyclic rings in CBZ-reactive patients (single data). **(C)** Chemical structures of ASMs and the ECBZ metabolite.

A previous study examining active cases of HLA-B*15:02-positive SJS (and one SJS/TEN) and CBZ-tolerant controls showed CBZ-induced CD8^+^ T cell cross-reactivity towards other tricyclic aromatic compounds (21). We wanted to determine whether T cell cross-reactivity can be achieved following reactivation of drug-induced T cells in our cohort of resolved HLA-B*15:02-positive SJS or TEN patients and a drug-tolerant control, where PBMCs were collected between 3-to-20 years post-reaction (**Table 1**). Here, *in vitro* expanded CBZ-induced T cell lines (generated for **Figure 1**) were restimulated in a 6-hour ICS assay with media alone (untreated), CBZ (25 μg/mL) or C1R.B*15:02 APCs in the absence or presence of drug (CBZ, ECBZ, OXC, PHT; 25 μg/mL) and T cell activation measured *via* IFNγ production, with statistical significance only observed between untreated and C1R.B*15:02+CBZ (p=0.111, one-way ANOVA with post-hoc Tukey’s multiple comparison test). We show that CD8^+^ T cells primed against the parent drug CBZ can cross-react towards other ASMs following restimulation with C1R.B*15:02+drug. As expected, restimulation in the presence of CBZ+APC generated the highest reactivity (mean ± SEM; 6.771 ± 2.220), followed by cross-reactivity towards OXC (3.014 ± 0.831) and ECBZ (2.943 ± 0.496) (**Figures 3A, B**). Whilst, minimal CD8^+^ T cell cross-reactivity was observed towards the aromatic drug PHT (1.657 ± 0.561), reflecting the more chemically diverse structure of PHT compared to the other more closely related compounds to CBZ (**Figure 3C**).

### CD8^+^ T Cells That Respond to CBZ Exhibit a High Degree of TCR Clonality

**Figure 4:**
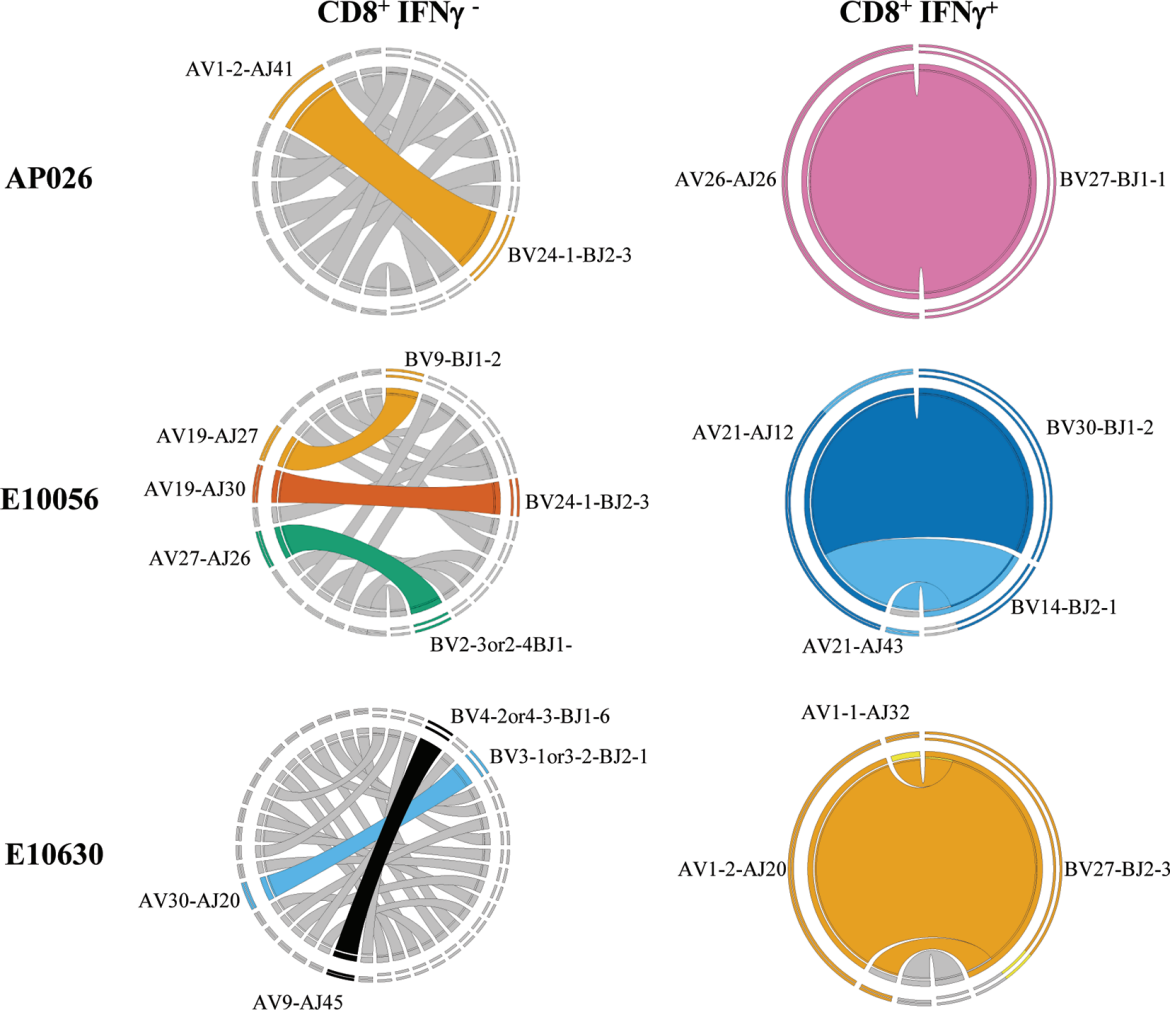
CBZ exposure drives TCR clonality. Representative Circos plots for drug-naïve individual (AP026) and resolved SJS (E10630) and TEN (E10056) patients showing dominant αβTCR clonotype pairings for both CD8^+^ IFNγ^-^ and CD8^+^ IFNγ^+^ T cell subsets. Circos plots were generated using an on-line feature based on concatenated pairings of TCRAVJ-CDR3A and TCRBVJ-CDR3B (46). Complete TCR sequencing is shown in **Supplementary Table 2**.

To evaluate whether the TCR repertoire of activated CD8^+^ T cells was remodeled following CBZ culture, we analyzed *in vitro* expanded HLA-B*15:02-positive PBMCs from two drug-naïve donors (AP022, AP026), five CBZ-induced SJS cases (T00016, T00024, E10076, E10314, E10630) and one CBZ-induced TEN case (E10056) (**Table 1**). CBZ-reactive T cell lines established from each individual were restimulated for 4-hours with C1R.B*15:02 APCs either in the absence (untreated) or presence of CBZ (treated, 25 μg/mL), with activated T cells detected by IFNγ secretion. T cells were segregated based on phenotype and activation status (non-activated: CD8^+^IFNγ^-^ or activated: CD8^+^IFNγ^+^), then single cell sorted for subsequent αβTCR repertoire analysis (**Supplementary Figure 2C**). Profiling of the activated CBZ-reactive CD8^+^ TCRs revealed a single major clonotype observed for the majority of individuals; AP022 (TRAV12-3/TRBV5-5; n=9/23 pairs), AP026 (TRAV26-1/TRBV27; 16/16), T00024 (TRAV4/TRBV3-1or3-2; 6/23), E10056 (TRAV21/TRBV30; 8/12) and E10630 (TRAV1-2/TRBV27; 18/24). Interestingly, AP026 and E10630 share the same TRBV27 usage (**Figure 4**, **Table 2**). Whilst, two major clonotypes were detected for T00016 (TRAV1-1/TRBV14; 4/23 and TRAV8-2 or 8-4/TRBV15; 4/23) and no clonality was demonstrated for E10076 or E10314 (HLA-B*15:02-negative donor). All TCR sequencing information is listed in **Supplementary Table 2**. We selected three CBZ-reactive CD8^+^ TCRs for further functional validation to understand antigen processing requirements for T cell stimulation (**Figure 4**, **Table 2**).

**Table 2:** αβTCR profiling of CBZ-reactive CD8^+^ T cells.

ID	Drug Reaction	TRAV	TRAJ	CDR3α	TRBV	TRBJ	CDR3β	Pair frequency (n)
AP026	Naïve	26-1	26	CIVRSLRDNYGQNFVF	27	1-1	CASRAGGNTEAFF	16/16
E10056	TEN	21	12	CAAKDGMDSSYKLIF	30	1-2	CAWLGAGKVDGYTF	8/12
E10630	SJS	1-2	20	CAAFGDYKLSF	27	2-3	CASSSLSGGWPDTQYF	18/24

### Clonotypic TCRs Exhibit Cross-Reactivity Towards CBZ Metabolites and Related ASMs

**Figure 5:**
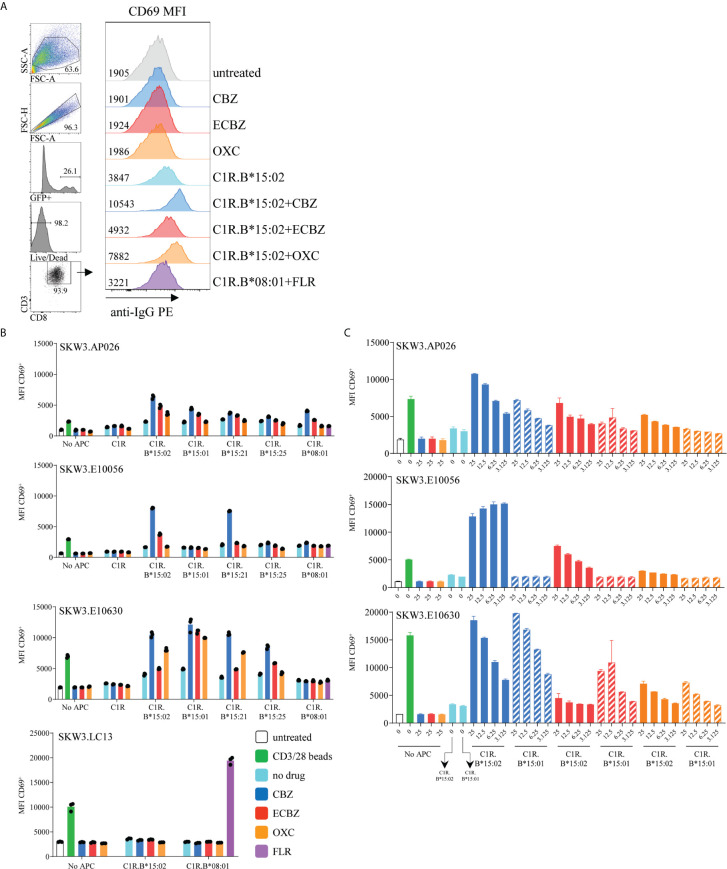
HLA-B*15:02-restricted CBZ-reactive TCRs recognize other drug-exposed HLA-B15 allomorphs. **(A)** CD69 upregulation assay and flow cytometric gating strategy to measure activated SKW3.TCR transfectants. Representative data shown from SKW3.E10630, with cell surface CD69 measured as geometric mean fluorescence intensity (MFI). **(B)** CBZ-reactive TCR lines (SKW3.AP026, SKW3.E10056, SKW3.E10630) and an EBV-specific irrelevant control (SKW3.LC13) were stimulated with APCs expressing different HLA-B15 allomorphs in the presence of tricyclic aromatic compounds, and the control C1R.B*08:01 presenting an irrelevant viral peptide (C1R.B*08:01+FLR) for 17-20 hours. Cells were then stained and data was acquired by flow cytometry (triplicate data, mean ± SEM). **(C)** Drug dose response titrations (3.125 to 25 μg/mL) were performed for each HLA-B15 allomorph prior to stimulation of CBZ-reactive TCR lines (SKW3.AP026, SKW3.E10056, SKW3.E10630) (duplicate data, mean ± SEM).

Retroviral transduction of selected CBZ-reactive CD8^+^ TCRs into the SKW3 reporter cell line enabled us to conduct cellular investigations of TCR recognition to confirm drug specificity without confounding background T-T presentation (SKW3 reporter cell lines are HLA-B15-negative). Here, TCR activation was determined by cell surface upregulation of CD69, with the shift in geometric MFI being measured by flow cytometry (**Figure 5A**). We next explored whether HLA allotypes associated with SJS/TEN risk (HLA-B*15:02, B*15:21; members of B75 serological subgroup) or non-risk (B*15:01, B*15:25; members of B62 serological subgroup) were able to stimulate CBZ-reactive TCRs in the presence of drug. A control APC presenting an irrelevant viral peptide (C1R.B*08:01/FLR) was also included. For SKW3.AP026, the greatest responses were observed for CBZ, ECBZ and OXC in the presence of C1R.B*15:02-positive cells. However, there does appear to be evidence of non-specific TCR drug-induced responsiveness across all HLA-B15 allotypes, as well as the HLA-B*08:01 control (**Figure 5B**, top panel). For SKW3.E10056, TCR recognition was highly restricted to HLA risk allotypes B*15:02 and B*15:21 and almost exclusively directed against the CBZ parent drug, with a small response shown for ECBZ in the presence of C1R.B*15:02 cells. Whilst for SKW3.E10630, the patterns observed for TCR recognition indicate a high degree of promiscuity towards risk and non-risk HLA-B15 allotypes across all drugs. Additionally, the hierarchy of drug recognition was altered by the presenting allotype, with risk allotype (HLA-B*15:02/B*15:21) mediated responses being highest for CBZ>OXC>ECBZ, whilst non-risk allotype (HLA-B*15:01/B*15:25) mediated responses showed an alternate hierarchy of CBZ>ECBZ>OXC. As expected, no SKW3.E10056 and SKW3.E10630 TCR activation towards the irrelevant control (C1R.B*08:01/FLR) was observed (**Figure 5B**, middle panels). Notably, none of the TCRs examined displayed responses to any of the drugs in the presence of the parental C1R cell line, which lacks HLA-A and -B expression. An irrelevant TCR control, SKW3.LC13, was also examined to demonstrate TCR specific recognition of C1R.B*08:01/FLR and non-activation by drug in the presence of C1R.B*08:01 or C1R.B*15:02 APCs (**Figure 5B**, lower panel).

To determine whether TCR recognition of the non-risk HLA allotype B*15:01 was drug dose dependent, the drug was titrated 4-fold from 25 μg/mL down to 3.125 μg/mL in the presence of APCs. For both SKW3.AP026 and SKW3.E10630, as anticipated TCR activation reduced in accordance with decreasing concentrations of drug. However, this effect was comparable in both the risk and non-risk HLA allotypes, B*15:02 and B*15:01, respectively. Whilst SKW3.E0056 showed remarkable specificity and sensitivity towards CBZ and decreasing responses towards the less immunogenic ECBZ, but only when presented by B*15:02-expressing APC (**Figure 5C**).

### SKW3.TCR Reporter Cells Are Activated *via* a Non-Covalent Drug Interaction

**Figure 6:**
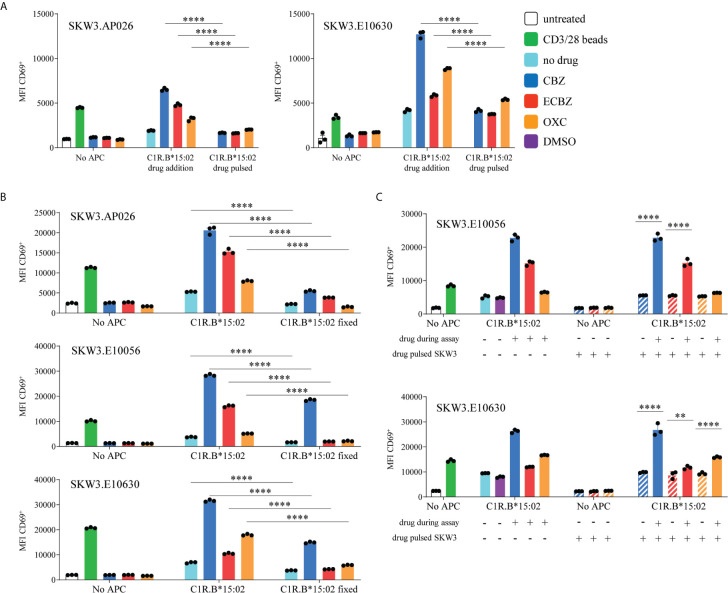
Features of SKW3.TCR activation. **(A)** CBZ-reactive TCR lines (SKW3.AP026, SKW3.E10630) were stimulated for 17-20 hours with C1R.B*15:02 either in the presence of drug throughout the assay (drug addition) or drug-pulsed overnight and then washed. Cells were then stained and data was acquired by flow cytometry (triplicate data, mean ± SEM). A significant decrease was observed when SKW3.TCR cells were stimulated with drug-pulsed C1R.B*15:02 (****p<0.0001; ANOVA with Tukey correction). **(B)** SKW3.TCR cells (AP026, E10056, E10630) were stimulated with either untreated or fixed C1R.B*15:02 for 17-20 hours. Cells were then stained and data was acquired by flow cytometry (triplicate data, mean ± SEM). Whilst a significant reduction in TCR activation was shown, a drug-reactive response was still observed (****p<0.0001; ANOVA with Tukey correction). **(C)** SKW3.E10056 and SKW3.E10630 cells were either untreated or pulsed with drug prior to stimulation with C1R.B*15:02 ± drug. TCR/drug interactions alone were unable to induce CD69 upregulation (****p<0.0001 and **p<0.005; ANOVA with Tukey correction).

We explored whether the SKW3.TCR cells were activated by the same non-covalent drug interactions as observed for *in vitro* expanded CBZ-induced T cell lines. Here, we tested the TCR transduced reporter cells capacity for activation when both C1R.B*15:02 and drug were present throughout the assay (drug addition), as well as when C1R.B*15:02 was pulsed overnight with drug and then washed prior to co-incubation. Both SKW3.AP026 (drug-naïve healthy control) and SKW3.E10630 (SJS patient) cell lines showed a significant decrease in TCR activation for C1R.B*15:02 drug-pulsed, compared to C1R.B*15:02 drug addition, across all three drugs tested (CBZ, ECBZ, OXC; p<0.0001 ANOVA with Tukey correction) (**Figure 6A**). These observations mirrored T cell line data (**Figures 1B**, **2A**) demonstrating a labile drug-HLA-B*15 interaction in keeping with non-covalent binding.

### SKW3.TCR Reporter Cells Do Not Require Intracellular Antigen Processing for Activation

We measured whether active antigen processing in APCs for *de novo* generation of peptide/HLA complexes was required for SKW3.TCR activation. Following fixation of C1R.B*15:02 with 1% paraformaldehyde, TCR activation was significantly reduced, compared to non-fixed C1R.B*15:02, but a notable drug-specific response was still observed for all three SKW3.TCR cell lines (**Figure 6B**, p<0.0001 ANOVA with Tukey correction). These data align with the nature of interaction between TCR/drug/HLA molecule not requiring active antigen processing pathways. Finally, we investigated whether the drug itself binds stably to the TCR. Here SKW3.E10056 and SKW3.E10630 cells were either untreated or pulsed with drug (and washed to remove unbound drug) prior to stimulation with C1R.B*15:02 in either the absence or presence of drug. Here, maximal TCR activation was only observed when TCR/drug/C1R.B*15:02 were present throughout the assay. Thus, potential TCR/drug interactions alone were assumed to lack the stability to be maintained without either maintenance of the drug in solution or stabilization by the HLA present on APC (**Figure 6C**, p<0.0001 and p<0.005 ANOVA with Tukey correction).

### Minimal Impact of CBZ/ECBZ/OXC on Peptide Binding Specificity of HLA-B*15:02

Previous analyses of the ligandome of HLA-B*15:02 from CBZ treated cells have observed co-purification of CBZ (22, 47). Furthermore, we previously described a minor alteration of the immunopeptidome reflected in conservation of anchor residue biases (P2, P9) of co-purified peptides, and modulation of residues across non-anchor positions. This earlier observation led us to postulate drug binding at a central position in the antigen-binding cleft (22). In contrast the metabolite ECBZ has been suggested to alter peptide binding to soluble HLA-B*15:02, through interaction in the region of the F-pocket (23). Given the responses of CBZ-reactive T cells to CBZ, ECBZ and OXC we sought to determine whether we could define common changes in peptide binding induced by these three small molecules that might shed light on the interaction. As previously, we utilized the C1R.B*15:02 cell line due to its demonstrated ability to present these molecules in an immunogenic fashion (**Figures 5**, **6**). Membrane-bound HLA molecules were extracted from 0.9-1.1 x 10^9^ cells through non-denaturing lysis and immunoaffinity purification, prior to acid elution of peptides, fractionation by RP-HPLC and LC-MS/MS analysis. Experiments were performed in duplicate for each of untreated and CBZ treated (25 μg/mL) cells, whilst single experiments for ECBZ and OXC treatment (25 μg/mL) conditions were performed to determine if ECBZ and OXC recapitulated observations for CBZ treatment.

As CBZ, ECBZ and OXC were observed to have distinct chromatographic retention times during RP-HPLC, aligned with fraction pools 10 (CBZ), 12 (ECBZ) and 13 (OXC), these specific pools were also analyzed by multiple reaction monitoring (MRM) to determine whether drug had co-purified with the HLA-B*15:02 during immunoaffinity purification. As anticipated, clear signal for CBZ was observed in pool 10 for CBZ treated samples at 36 minutes, for ECBZ in pool 12 of ECBZ treated samples at 28 minutes, and for OXC in pool 13 for OXC treated samples at 30 minutes (**Supplementary Figure 3**). Despite identical masses and monitored transitions, OXC and ECBZ are distinguished by distinct retention times and transition hierarchies (**Supplementary Figure 3B** *vs* **3C**). In the ECBZ sample, a second peak was also observed at 21 minutes (**Supplementary Figure 3B** **)**. Evolution of this second, earlier, peak was also seen over increased time in the autosampler for ECBZ prepared as a comparator. Subsequent analysis suggested that ECBZ is unstable over time in the loading conditions used for LC-MS analysis (2% ACN, 0.1% FA), with an increase in signal for a mass consistent with the hydrolysis product (10,11-dihydroxycarbamazepine, m/z = 271.1+) co-eluting with the earlier peak (data not shown). It is therefore hypothesized that the earlier peak is 10,11-dihydroxycarbamazepine, some of which undergoes dehydration during electrospray ionization to regenerate ECBZ.

**Figure 7:**
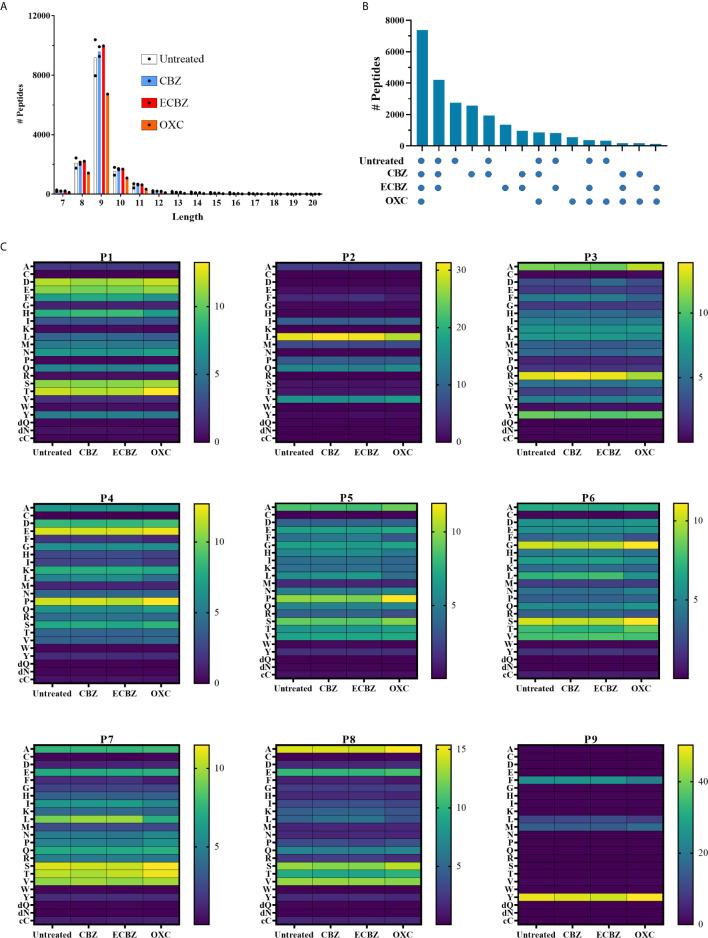
Binding specificity of HLA-B*15:02 is not overtly perturbed by CBZ, ECBZ or OXC. C1R.B*15:02 were cultured for 48 hours in the presence of 25 μg/mL CBZ, ECBZ or OXC, or without drug. HLA class I molecules were isolated by immunoaffinity purification and the bound peptides/drugs eluted and analyzed by LC-MS/MS to characterize the HLA-B*15:02 immunopeptidome under these conditions. Experiments were performed in duplicate for untreated and CBZ treatment conditions, and single experiments were performed for ECBZ and OXC treatment conditions. **(A)** Shows the length distribution of peptides identified in each condition with points representing the number of peptides in individual data sets, and bars representing the mean number of the peptides from duplicate experiments. **(B)** To identify the similarity between the conditions, peptides identified in duplicate experiments were combined and the number of peptides overlapping between the conditions depicted as an upset plot. Points below the graph depict the conditions to which the peptides are common. **(C)** The proportion of 9mers in each condition (mean for duplicate experiments) possessing a given amino acid at P1-P9 are depicted as heat maps. Individual scale bars are provided per position. Amino acids are shown using the single letter code, with the addition of dQ and dN [representing deamidated Gln(Q) and Asn(N)], and cC (representing Cysteinylation at Cys).

Analysis of the eluted peptides identified more than 9900 peptides of 7-20 residues per data set (**Figure 7A**) as described in the Materials and Methods. Over 24,000 peptides were identified in total, with more than 7,000 peptides identified in all treatments (**Figure 7B**). Peptides were predominantly 9 amino acids in length, which were heavily biased towards Leu (~30%)>Val (~16%)>Gln (~14%) at position 2 of the peptide, and Tyr (~48%)>Phe (~24%)>Met (~15%)>Leu (~10%) at position 9 (**Figure 7C**). Proline was observed at P4 and P5 in approximately 12% and 10% of peptides respectively, whilst Ser, Thr and Val were often seen in positions 5-8 of the peptide (**Figure 7C**). Only minor differences in amino acid prevalence were observed between individual treatments and the untreated control, and no common perturbation of residue preference was noted for the three molecules when considering the global immunopeptidome as might be expected from their cross-reactivity (**Supplementary Figure 4**). Due to previous reports of increased prevalence of deamidated peptides bound to HLA-B*15:02 after treatment with ECBZ, particularly at P4 (23), we considered deamidated Asn and Gln separately from their native form in motif analysis. However, deamidated peptides were observed to be rare in our analysis (**Supplementary Figure 5**). We previously observed minor perturbations of non-anchor residues when considering peptides uniquely identified in HLA-I purifications of CBZ treated C1R.B*15:02 as compared to untreated cells, in analyses based on a smaller dataset (<2000 peptides/condition) (22). The current analysis incorporates more than 9900 peptides (>6000 nonameric peptides) per experiment, and more than 18,000 peptides (>11,000 nonamers) per untreated and CBZ treated conditions when replicates are combined. We therefore again determined the peptides that were common (identified in at least 1 replicate of each condition) and unique (identified in at least 1 replicate of the condition, but never in the compared condition). We identified more than 14,000 peptides (>9000 nonamers) common to untreated and CBZ treatment conditions, with 4246 (2126 nonamers) and 3871 (2129 nonamers) peptides identified in untreated only (untreated unique) and CBZ treatment only (CBZ unique), respectively. Focusing on the nonamer peptides, as anticipated we saw similar anchor preferences to the whole data set analysis. As observed previously, there were some variations across the backbone of the peptide (**Supplementary Figures 6**), but few were recapitulated in similar analyses of ECBZ and OXC compared to untreated cells (**Supplementary Figures 7**, **8**), and overall only 2738 (1211 nonamers) were only identified in the untreated condition (when compared to all drug treatment conditions) (**Figure 7B**). It is therefore difficult to discern a compelling common drug induced motif across the three treatments, although a slight reduction in acidic residues at P1 and tyrosine at P3 were commonly observed (**Supplementary Figures 6**–**8**), as noted previously for CBZ (22). A subtle increase in Pro at P2 was also observed in peptides uniquely identified in drug treatments, although Leu remained the dominant anchor residue.

## Discussion

Adaptive immune recognition of a foreign entity by T cells relies on two signals; (1) interaction between an immunogenic antigen/HLA complex and the TCR and (2) co-stimulation provided by engagement of B7 (*i.e.* CD80/CD86) on APCs and CD28 expressed on T cells. The ensuing response drives expansion and proliferation of T cells containing an armory of effector functions, including pro-inflammatory cytokine production and release of cytotoxic molecules, that eliminate pathogen‐infected or neoplastic cells. Once the elimination process is completed, attrition of effector T cells leads to the establishment of a memory T cell pool to combat secondary encounters with the same immunogenic antigen/HLA complex which lacks the necessity for signal 2 to allow rapid deployment. Whilst T cell-mediated immune responses are critical in controlling initial and subsequent challenges, responses against innocuous stimuli sometimes occur and are associated with autoimmunity and allergy, including DHRs.

The impacts of ASM-induced T cell-mediated DHRs have been well reported [comprehensively reviewed (6, 48)]. Key insights have been drawn from investigation of three main cohorts; ASM-induced DHRs [spanning mild (MPE), moderate (DRESS) and severe (SJS and/or TEN) cases], drug tolerant patients and drug-naïve healthy donors. Comparison between these cohorts has provided critical information regarding the culprit ASM (*e.g*. CBZ, OXC, PHT, lamotrigine) as well as the association of HLA allotype (*e.g.* B*15:02, A*31:01) and ethnicity (*e.g.* Han Chinese ancestry) on DHR outcomes. Importantly, immunopathological examinations during the active phase of the hypersensitivity reaction has demonstrated a dominant role for CD8^+^ T cells and the involvement of key cytolytic (*e.g.* granulysin, perforin, granzyme) and proinflammatory cytokines (*e.g.* IFNγ, macrophage inflammatory protein-1α) (21, 28, 31, 49–52).

In this study, we examined long-lived T cell reactivity profiles of resolved CBZ-induced SJS (and one TEN) patients, ranging from 3 to 20 years post-active disease. In particular, we were interested in determining whether CBZ-reactive memory T cells activated upon re-exposure to drug showed similar profiles to those reported in active disease, considering effector functions, TCR repertoire clonality, mode of drug recognition and cross-reactivity towards other ASMs.

CBZ-reactive T cells derived from SJS or TEN patient PBMCs were expanded for 2 weeks *in vitro* following a single round of CBZ exposure. The outgrown bulk T cell culture was then restimulated with drug either in the absence or presence of an HLA-B*15:02-expressing APC, and immunophenotypic properties of CBZ-reactive effector T cells measured by flow cytometry. We showed that these restimulated T cells were primarily restricted to the CD8^+^ T cell lineage and were capable of Th1 pro-inflammatory cytokine production (IFNγ > TNF), aligning with reported studies of SJS/TEN patients during active disease (21, 28). For drug-naïve healthy donors, 2-3 rounds of drug exposure (CBZ or ECBZ) were required for *in vitro* priming and expansion of drug-reactive T cells. Whilst, CBZ- and ECBZ-reactive CD8^+^ T cells were observed by day 40 in all individuals, the ECBZ metabolite had lower levels of immune responsiveness (with IFNγ > TNF) compared to the parent drug, CBZ.

A common complication of prescribed ASMs is that individuals who have experienced a DHR are at higher risk of developing the same, if not, a more severe form of DHR with an alternate ASM. Indeed, structural similarities exhibited by aromatic ASMs (*i.e.* CBZ, OXC, PHT, lamotrigine) provide a framework for cross-reactive T cell recognition that perpetuates secondary reactions in between 13-80% of DHR patients (53–56). This observation is supported by cellular investigations in SJS/TEN patients with CBZ-induced T cells (generated following 4-5 rounds of *in vitro* culture) exhibiting cross-reactivity towards other aromatic compounds such as ECBZ, OXC, lamotrigine and eslicarbazepine (21). Whilst, non-aromatic ASMs (*i.e.* valproic acid and gabapentin) are not generally associated with DHRs (57). In this study, we demonstrated that CBZ-reactive T cells (generated following a single *in vitro* drug exposure) cross-react with HLA-B*15:02^+^ APCs in the presence of aromatic ASMs ECBZ and OXC. The majority of patients showed higher levels of cross-reactivity towards the ECBZ metabolite, albeit one individual (E10630) demonstrated greater reactivity towards OXC. These data suggest an immune hierarchy of T cell cross-reactivity towards ASMs is likely driven by the common tricyclic ring structure between CBZ, ECBZ and OXC, but modulated by different side groups. These observations demonstrate that resolved SJS and TEN patients, many years post-DHR, can still vigorously respond to alternate aromatic ASMs, and that T cell immune monitoring should be undertaken when considering different treatment options.

We next examined the HLA-B*15:02-restricted TCR repertoire of CBZ-reactive CD8^+^ T cells in two drug-naïve individuals and six SJS or TEN patients. In most cases, a single major TCR clonotype was observed, which supports previous findings of focused TCR usage in SJS/TEN patients (28). However, most of these TCR repertoires showcased private specificities, with only two individuals (AP026 and E10630) sharing the same TRBV27 usage, but lacking common CDR3 regions. These findings diverge from previous reports of a more public TCR usage within the HLA-B*15:02-restricted CBZ-induced TCR repertoire in SJS and/or TEN patients; that exhibited dominant Vα and Vβ clonotypes (28) and a public αβTCR CDR3 sequence (24). Differences in TCR repertoire may also be attributed to either the site of origin for CBZ-reactive T cells [*i.e.* blister fluid/cells vs PBMCs [this study)] and/or collection of biospecimens during active or resolved (this study) disease states. It is also important to acknowledge reported biases associated with the *in vitro* expansion of T cell lines (29, 58), with the likelihood of different TCR clonotypes being expressed by drug-naïve and drug-experienced individuals, as well as their localization at different sites of pathology (i.e. blister fluid vs PBMCs). In particular, the study by Pan et al. (24) could not recapitulate TCR clonotype findings reported by Ko et al. (28) in the PBMC and blister fluid of SJS/TEN patients. Although, we have previously shown in anti-viral TCRs that a bias was not induced between *ex vivo* PBMCs and day 14 *in vitro* T cell cultures (59).

Limited patient biospecimen availability makes it difficult to conduct a multitude of cellular assays, even with *in vitro* expansion of T cell lines and clones. Here we take advantage of a known reporter system utilizing αβTCR deficient SKW3 cells (41, 60) to examine drug-induced HLA-restricted T cell promiscuity and the mode of drug presentation. Single major TCR clonotypes, for one drug-naïve individual (AP026) and two patients (E10056, E10630), were cloned into the SKW3 reporter cell line. Firstly, we investigated whether these TCRs demonstrated HLA-B15 specificity towards SJS or TEN risk (B75 subgroup) and non-risk (B62 subgroup) allotypes (21). Interestingly, we observed highly restricted TCR recognition for E10056 towards B75 subgroup members B*15:02 and B*15:21, whilst both AP026 and E10630 showed high degree of cross-reactivity towards risk and non-risk HLA-B*15 allotypes. This observation was apparent for all HLA-B15 APCs in the presence of CBZ, ECBZ or OXC in a dose dependent fashion. Furthermore, no responses were observed in the presence of HLA-B-negative C1R cells. These observations suggest that non-risk HLA-B15 molecules may also support the ligation of certain CBZ-reactive TCRs, implying the association of SJS or TEN with HLA-B*15:02, but not HLA-B*15:01, is not as simple as the ability of the drug to uniquely interact with one HLA allomorph but not another.

Secondly, we examined whether ASMs stimulated SKW3.TCR reporter cells using the same mechanisms required for T cell lines. As observed by others (21, 49, 52), we found it was essential to maintain soluble drug continuously within the assay for TCR activation, consistent with a non-covalent and labile interaction between the drug and peptide/HLA and/or TCR complex. Additionally, we showed that fixation of APCs did not abrogate drug-induced TCR activation, which aligns with the interaction occurring with pre-existing peptide/HLA complexes, hence not requiring additional antigen processing. Finally, we showed that TCR/drug interactions were not maintained on washing of the reporter cells at physiological pH suggesting any interactions lack sufficient stability to be maintained in the absence of the peptide/HLA complex. Whilst it has been suggested that binding can occur directly with a public CBZ-specific αβTCR in the absence of the peptide/HLA complex (24), we only observed reporter cell activation in the presence of drug treated HLA-B*15-positive APCs. Hence, the presence of peptide/HLA, and TCR are necessary for maximal functional coordination of the drug interaction. Collectively, these data validate the use of SKW3.TCR reporter cells as an immunological tool for DHR cellular investigations and confirm that non-covalent and labile drug/TCR/peptide/HLA interactions in the absence of new peptide/HLA generation underpin CBZ-induced DHRs.

In line with the inability of drug pulsed C1R.B*15:02 to stimulate reporter cell responses, modulation of the immunopeptidome by CBZ and its derivatives was subtle. This is distinct from the interaction of abacavir and HLA-B*57:01, where stable, non-covalent binding within the antigen binding cleft causes a perturbation of self-peptide presentation, initiated predominantly in the ER, and alteration of the PΩ anchor preference of abacavir occupied HLA-B*57:01 molecules (22, 26, 27). We previously observed subtle modulation of non-anchor residues of peptides bound to HLA-B*15:02 through comparison of a smaller dataset of HLA-B*15:02 ligands (<2000 peptides per condition). With the current larger dataset (>9900 peptides per condition) and comparison to cross-reactive molecules ECBZ and OXC not all changes were recapitulated, although a subtle reduction in acidic amino acids at P1 and tyrosine at P3 were consistent for peptides unique to drug treatments (22). This is not to say there are not HLA/peptide/drug complexes formed but the mechanism for their formation clearly follows a different trajectory to that of HLA-B*57:01/abacavir/peptide complexes. Interestingly, our data contrasted a recent report that ECBZ alters peptide presentation by soluble HLA-B*15:02 (23). Whether this is due to differences between soluble and membrane-bound HLA such as reduced interaction with the peptide loading complex, or the workflow used (*e.g.* the necessity for use of detergents to extract membrane-bound HLA, peptide elution conditions), which might impact complex stability, remains to be clarified.

As described previously, CBZ and ECBZ were detected in eluates from affinity purified HLA class I molecules of drug treated cells (22, 23, 47), an observation that was extended to OXC. This residual drug is maintained despite washing of the source cells, as well as the immunoaffinity purified HLA class I molecules, at physiological pH, a process shown to abrogate APC immunogenicity in functional experiments. Given the rapid titration of responses to CBZ, ECBZ and OXC, it is possible that whilst washing reduces the number of peptide/HLA/drug complexes below the threshold for activation, some level of binding is maintained. Regardless, failure to identify a distinct peptide motif in drug treated cells and the inability of either the peptide/HLA or TCR alone to maintain sufficient drug interaction for immunogenicity on removal of soluble drug, collectively suggest that CBZ and related compounds form a tripartite interaction with peptide/HLA/TCR. Furthermore, differences in recognition hierarchies of CBZ, ECBZ and OXC by the three TCRs investigated suggest the epoxide (ECBZ) and ketone (OXC) more prominently impact TCR ligation as opposed to interaction with the HLA, thus may be oriented towards the TCR.

In conclusion, this study confirms the presence of long-lived immunological effects in resolved CBZ-induced SJS and TEN patients, which are characterized by a highly clonal drug-reactive CD8^+^ TCR repertoire. Furthermore, using a combination of the definition of the functional requirements for TCR engagement using transduced reporter cell lines and detailed study of the impact of CBZ, ECBZ and OXC on HLA-B*15:02 peptide presentation, our work supports the hypothesis that peptide/HLA and TCR are required for drug interactions able to elicit TCR activation.

## Data Availability Statement 

The datasets presented in this study can be found in online repositories. The names of the repository/repositories and accession number(s) can be found below: https://www.ebi.ac.uk/pride/archive/, PXD023545.

## Ethics Statement 

The studies involving human participants were reviewed and approved by Joint Chinese University of Hong Kong-New Territories East Cluster Clinical Research Ethics Committee (Hong Kong; CRE-2006.203 for patients), Monash University (Victoria, Australia; HREC-4717 for healthy individuals), and the Australian Bone Marrow Donor Registry (New South Wales, Australia; 2013/04 for healthy individuals). The patients/participants provided their written informed consent to participate in this study.

## Author Contributions

NM, PI, JR, JV, and AP designed experiments. NM, PI, JL, HF, and ZH performed experiments. LH, PK, and AP provided reagents and/or patient samples. NM, PI, JL, and AP analyzed data. NM, PI, and AP wrote the manuscript. All authors contributed to the article and approved the submitted version.

## Funding

PI was supported by a National Health and Medical Research Council of Australia (NHMRC) Early Career Fellowship (1072159, 2014-2017) and a Monash University Faculty of Medicine, Nursing and Health Sciences Senior Postdoctoral Fellowship (2020). LH was recipient of Melbourne International Research Scholarship and Melbourne International Fee Remission Scholarship. JR is supported by Australian Research Council Australia Laureate Fellowship. PK is supported by a Medical Research Future Fund Fellowship (MRF1136427). AP is supported by a NHMRC Principal Research Fellowship (1137739). We acknowledge funding support from a NHMRC Project grants 1103979 (to PK) and 1122099 (to AP and JV).

## Conflict of Interest

*The authors declare that the research was conducted in the absence of any commercial or financial relationships that could be construed as a potential conflict of interest.*

## Supplementary Material

Supplementary Figure 1 HLA staining of Antigen Presenting Cells and SKW3.TCR expression. C1R Parental and transfected cells expressing different HLA-B15 allotypes were stained with primary antibodies **(A)** anti-human pan HLA-I (W6/32 hybridoma) and **(B)** anti-human HLA-Bw6 (HB152 hybridoma) followed by a goat anti-mouse IgG PE secondary antibody. A secondary (2°) antibody alone control was used for background staining. **(C)** All four SKW3 cell lines were periodically tested for TCR cell surface expression. A total of 30,000 cells were acquired on LSRII flow cytometer (BD) and data was analyzed using FlowJo software (version 10, BD).

Supplementary Figure 2
*In vitro* expansion of CBZ-reactive T cells and cell sorting for αβTCR repertoire analysis. **(A)** Drug-reactive T cells were outgrown following 14 days of PBMC stimulation with 25 μg/mL CBZ. Day 14 T cells were restimulated for 6 hours with C1R cells expressing different HLA-B15 allotypes in the presence or absence of 25 μg/mL CBZ. T cell phenotype and activation was then measured by flow cytometry. **(B)** Gating strategy shown is representative data from SJS patient E10630. A maximum of 50,000 lymphocytes were acquired on LSRII flow cytometer (BD) and data was analyzed using FlowJo software (version 10, BD). **(C)** Day 14 CBZ-reactive T cells were restimulated with C1R.B*15:02 in either the absence (negative control) or presence of 25 μg/mL CBZ for 4 hours. Activated CD8+ T cells were detected using an IFNγ secretion assay. Single cell sorting of both CD8^+^ IFNγ^-^ and CD8^+^ IFNγ^+^ T cell subsets for TCR repertoire analysis were performed using an Influx flow cytometer (BD).

Supplementary Figure 3 Drug molecules co-purify with HLA-B*15:02. **(A-C)** Show raw intensities for transitions monitoring **(A)** CBZ in pool 10 of CBZ replicate 1, **(B)** ECBZ in pool 12 of ECBZ, **(C)** OXC in pool 13 of OXC. **(D-F)** summarize the normalized signal for CBZ **(D)**, ECBZ (separated into the signal at 21 minutes (RT 21) and 28 minutes (RT 28) **(E)** and OXC **(F)** in pools 10, 12 and 13, respectively.

Supplementary Figure 4 Deviations in amino acid prevalence observed between treatments are minimal. Points represent differences between the amino acid prevalence at each position for drug/metabolite treatments as compared to the untreated control (mean of duplicate experiments). For CBZ, points are the difference of the mean of duplicate data sets with error bars showing standard error of the difference (calculated using Welch t-test).

Supplementary Figure 5 No major alteration of the contribution of deamidated peptides to the HLA-B*15:02 immunopeptidome by drug treatment. Graphs show the percentage of 9mer peptides annotated as containing native Glutamine (top left), native Asparagine (top right), deamidated Glutamine (bottom left) and deamidated asparagine (bottom right) at each position of the peptide. For untreated and CBZ treatment, values are the mean of two replicate experiments, error bars show the standard deviation. ECBZ and OXC treatment are singlet values.

Supplementary Figure 6 Amino acid prevalence for 9mer peptides unique to CBZ treatment when compared to control conditions. Combined lists of peptides identified in at least 1 replicate of the control condition (untreated) and CBZ treatment were generated and compared to generate 3 categories; untreated unique – identified in at least one replicate of the untreated control, but not in either replicate of CBZ treatment, common – identified in at least one replicate of the untreated control and at least one replicate of the CBZ treatment, and CBZ unique – identified in neither replicate of the untreated control, but in at least one replicate of CBZ treatment. Bar graphs show the percentage of 9mer peptides possessing a given amino acid at each position of the peptide per category. n is the number of 9mer peptides in the category. Arrows indicate residues discussed in the text.

Supplementary Figure 7 Amino acid prevalence for 9mer peptides unique to ECBZ treatment when compared to control conditions. A combined list of peptides identified in at least 1 replicate of the control condition (untreated) was generated and compared to the ECBZ treatment data set to generate 3 categories; untreated unique – identified in at least one replicate of the untreated control, but not in ECBZ treatment, common – identified in at least one replicate of the untreated control and in the ECBZ treatment, and ECBZ unique – identified in neither replicate of the untreated control, but in ECBZ treatment. Bar graphs show the percentage of 9mer peptides possessing a given amino acid at each position of the peptide per category. n is the number of 9mer peptides in the category. Arrows indicate residues discussed in the text.

Supplementary Figure 8 Amino acid prevalence for 9mer peptides unique to OXC treatment when compared to control conditions. A combined list of peptides identified in at least 1 replicate of the control condition (untreated) was generated and compared to the OXC treatment data set to generate 3 categories; untreated unique – identified in at least one replicate of the untreated control, but not in OXC treatment, common – identified in at least one replicate of the untreated control and in the OXC treatment, and OXC unique – identified in neither replicate of the untreated control, but in OXC treatment. Bar graphs show the percentage of 9mer peptides possessing a given amino acid at each position of the peptide per category. n is the number of 9mer peptides in the category. Arrows indicate residues discussed in the text.

## Abbreviations

APC, antigen presenting cell; ASM, antiseizure medication; CBZ, carbamazepine; DRESS, drug reaction with eosinophilia and systemic symptoms; DHR, drug hypersensitivity reaction; HLA, human leukocyte antigen; MPE, maculopapular exanthema; TCR, T cell receptor; PBMC, peripheral blood mononuclear cell; SJS, Stevens-Johnson syndrome; TEN, toxic epidermal necrolysis.

## References

[1] Micheletti RG Chiesa-Fuxench Z Noe MH Stephen S Aleshin M Agarwal A. Stevens-Johnson Syndrome/Toxic Epidermal Necrolysis: A Multicenter Retrospective Study of 377 Adult Patients from the United States. J Invest Dermatol (2018) 138(11):2315–21. 10.1016/j.jid.2018.04.027 29758282

[2] Roujeau JC Stern RS. Severe Adverse Cutaneous Reactions to Drugs. New Engl J Med (1994) 331(19):1272–85. 10.1056/NEJM199411103311906 7794310

[3] De A Rajagopalan M Sarda A Das S Biswas P. Drug Reaction with Eosinophilia and Systemic Symptoms: An Update and Review of Recent Literature. Indian J Dermatol (2018) 63(1):30–40. 10.4103/ijd.IJD_582_17 29527023 PMC5838752

[4] Marson AG Al-Kharusi AM Alwaidh M Appleton R Baker GA Chadwick DW. The SANAD study of effectiveness of carbamazepine, gabapentin, lamotrigine, oxcarbazepine, or topiramate for treatment of partial epilepsy: an unblinded randomised controlled trial. Lancet (2007) 369(9566):1000–15. 10.1016/S0140-6736(07)60460-7 PMC2080688 17382827

[5] Mockenhaupt M Messenheimer J Tennis P Schlingmann J. Risk of Stevens-Johnson syndrome and toxic epidermal necrolysis in new users of antiepileptics. Neurology (2005) 64(7):1134–8. 10.1212/01.WNL.0000156354.20227.F0 15824335

[6] Mullan KA Anderson A Illing PT Kwan P Purcell AW Mifsud NA. HLA-associated antiepileptic drug-induced cutaneous adverse reactions. HLA (2019) 93(6):417–35. 10.1111/tan.13530 30895730

[7] Chung WH Hung SI Hong HS Hsih MS Yang LC Ho HC. Medical genetics: a marker for Stevens-Johnson syndrome. Nature (2004) 428(6982):486. 10.1038/428486a 15057820

[8] Mehta TY Prajapati LM Mittal B Joshi CG Sheth JJ Patel DB. Association of HLA-B*1502 allele and carbamazepine-induced Stevens-Johnson syndrome among Indians. Indian J Dermatol Venereol Leprol (2009) 75(6):579–82. 10.4103/0378-6323.57718 19915237

[9] Tangamornsuksan W Chaiyakunapruk N Somkrua R Lohitnavy M Tassaneeyakul W. Relationship between the HLA-B*1502 allele and carbamazepine-induced Stevens-Johnson syndrome and toxic epidermal necrolysis: a systematic review and meta-analysis. JAMA Dermatol (2013) 149(9):1025–32. 10.1001/jamadermatol.2013.4114 23884208

[10] Tassaneeyakul W Tiamkao S Jantararoungtong T Chen P Lin SY Chen WH. Association between HLA-B*1502 and carbamazepine-induced severe cutaneous adverse drug reactions in a Thai population. Epilepsia (2010) 51(5):926–30. 10.1111/j.1528-1167.2010.02533.x 20345939

[11] Ikeda H Takahashi Y Yamazaki E Fujiwara T Kaniwa N Saito Y. HLA class I markers in Japanese patients with carbamazepine-induced cutaneous adverse reactions. Epilepsia (2010) 51(2):297–300. 10.1111/j.1528-1167.2009.02269.x 19694795

[12] Kaniwa N Saito Y Aihara M Matsunaga K Tohkin M Kurose K. HLA-B*1511 is a risk factor for carbamazepine-induced Stevens-Johnson syndrome and toxic epidermal necrolysis in Japanese patients. Epilepsia (2010) 51(12):2461–5. 10.1111/j.1528-1167.2010.02766.x 21204807

[13] Kim SH Lee KW Song WJ Kim SH Jee YK Lee SM. Carbamazepine-induced severe cutaneous adverse reactions and HLA genotypes in Koreans. Epilepsy Res (2011) 97(1-2):190–7. 10.1016/j.eplepsyres.2011.08.010 21917426

[14] Shi YW Min FL Qin B Zou X Liu XR Gao MM. Association between HLA and Stevens-Johnson syndrome induced by carbamazepine in Southern Han Chinese: genetic markers besides B*1502? Basic Clin Pharmacol Toxicol (2012) 111(1):58–64. 10.1111/j.1742-7843.2012.00868.x 22348435

[15] Genin E Chen DP Hung SI Sekula P Schumacher M Chang PY. HLA-A*31:01 and different types of carbamazepine-induced severe cutaneous adverse reactions: an international study and meta-analysis. Pharmacogenom J (2014) 14(3):281–8. 10.1038/tpj.2013.40 24322785

[16] Hsiao YH Hui RC Wu T Chang WC Hsih MS Yang CH. Genotype-phenotype association between HLA and carbamazepine-induced hypersensitivity reactions: strength and clinical correlations. J Dermatol Sci (2014) 73(2):101–9. 10.1016/j.jdermsci.2013.10.003 24268988

[17] Hung SI Chung WH Jee SH Chen WC Chang YT Lee WR. Genetic susceptibility to carbamazepine-induced cutaneous adverse drug reactions. Pharmacogenet Genomics (2006) 16(4):297–306. 10.1097/01.fpc.0000199500.46842.4a 16538176

[18] McCormack M Alfirevic A Bourgeois S Farrell JJ Kasperaviciute D Carrington M. HLA-A*3101 and carbamazepine-induced hypersensitivity reactions in Europeans. N Engl J Med (2011) 364(12):1134–43. 10.1056/NEJMoa1013297 PMC3113609 21428769

[19] Ozeki T Mushiroda T Yowang A Takahashi A Kubo M Shirakata Y. Genome-wide association study identifies HLA-A*3101 allele as a genetic risk factor for carbamazepine-induced cutaneous adverse drug reactions in Japanese population. Hum Mol Genet (2011) 20(5):1034–41. 10.1093/hmg/ddq537 21149285

[20] Mockenhaupt M Wang CW Hung SI Sekula P Schmidt AH Pan RY. HLA-B*57:01 confers genetic susceptibility to carbamazepine-induced SJS/TEN in Europeans. Allergy (2019) 74(11):2227–30. 10.1111/all.13821 30972788

[21] Wei CY Chung WH Huang HW Chen YT Hung SI. Direct interaction between HLA-B and carbamazepine activates T cells in patients with Stevens-Johnson syndrome. J Allergy Clin Immunol (2012) 129(6):1562–9 e5. 10.1016/j.jaci.2011.12.990 22322005

[22] Illing PT Vivian JP Dudek NL Kostenko L Chen Z Bharadwaj M. Immune self-reactivity triggered by drug-modified HLA-peptide repertoire. Nature (2012) 486(7404):554–8. 10.1038/nature11147 22722860

[23] Simper GS Ho GT Celik AA Huyton T Kuhn J Kunze-Schumacher H. Carbamazepine-Mediated Adverse Drug Reactions: CBZ-10,11-epoxide but Not Carbamazepine Induces the Alteration of Peptides Presented by HLA-B *15:02. J Immunol Res (2018) 2018:5086503. 10.1155/2018/5086503 30302345 PMC6158965

[24] Pan RY Chu MT Wang CW Lee YS Lemonnier F Michels AW. Identification of drug-specific public TCR driving severe cutaneous adverse reactions. Nat Commun (2019) 10(1):3569. 10.1038/s41467-019-11396-2 31395875 PMC6687717

[25] Illing PT Mifsud NA Purcell AW. Allotype specific interactions of drugs and HLA molecules in hypersensitivity reactions. Curr Opin Immunol (2016) 42:31–40. 10.1016/j.coi.2016.05.003 27261882

[26] Ostrov DA Grant BJ Pompeu YA Sidney J Harndahl M Southwood S. Drug hypersensitivity caused by alteration of the MHC-presented self-peptide repertoire. Proc Natl Acad Sci USA (2012) 109(25):9959–64. 10.1073/pnas.1207934109 PMC3382472 22645359

[27] Norcross MA Luo S Lu L Boyne MT Gomarteli M Rennels AD. Abacavir induces loading of novel self-peptides into HLA-B*57: 01: an autoimmune model for HLA-associated drug hypersensitivity. AIDS (2012) 26(11):F21–F9. 10.1097/QAD.0b013e328355fe8f PMC4155923 22617051

[28] Ko TM Chung WH Wei CY Shih HY Chen JK Lin CH. Shared and restricted T-cell receptor use is crucial for carbamazepine-induced Stevens-Johnson syndrome. J Allergy Clin Immunol (2011) 128(6):1266–76 e11. 10.1016/j.jaci.2011.08.013 21924464

[29] Koning D Costa AI Hasrat R Grady BP Spijkers S Nanlohy N. In vitro expansion of antigen-specific CD8(+) T cells distorts the T-cell repertoire. J Immunol Methods (2014) 405:199–203. 10.1016/j.jim.2014.01.013 24512815

[30] Xiong H Wang L Jiang M Chen S Yang F Zhu H. Comprehensive assessment of T cell receptor beta repertoire in Stevens-Johnson syndrome/toxic epidermal necrolysis patients using high-throughput sequencing. Mol Immunol (2019) 106:170–7. 10.1016/j.molimm.2019.01.002 30623817

[31] Lichtenfels M Farrell J Ogese MO Bell CC Eckle S McCluskey J. HLA restriction of carbamazepine-specific T-Cell clones from an HLA-A*31:01-positive hypersensitive patient. Chem Res Toxicol (2014) 27(2):175–7. 10.1021/tx400460w 24476427

[32] Cheung YK Cheng SH Chan EJ Lo SV Ng MH Kwan P. HLA-B alleles associated with severe cutaneous reactions to antiepileptic drugs in Han Chinese. Epilepsia (2013) 54(7):1307–14. 10.1111/epi.12217 23692434

[33] Shi YW Min FL Zhou D Qin B Wang J Hu FY. HLA-A*24:02 as a common risk factor for antiepileptic drug-induced cutaneous adverse reactions. Neurology (2017) 88(23):2183–91. 10.1212/WNL.0000000000004008 PMC5467955 28476759

[34] Zemmour J Little AM Schendel DJ Parham P. The HLA-A,B “negative” mutant cell line C1R expresses a novel HLA-B35 allele, which also has a point mutation in the translation initiation codon. J Immunol (1992) 148(6):1941–8. 1541831

[35] Storkus WJ Howell DN Salter RD Dawson JR Cresswell P. NK susceptibility varies inversely with target cell class I HLA antigen expression. J Immunol (1987) 138(6):1657–9. 3819393

[36] Mifsud NA Nguyen THO Tait BD Kotsimbos TC. Quantitative and functional diversity of cross-reactive EBV-specific CD8+ T cells in a longitudinal study cohort of lung transplant recipients. Transplantation (2010) 90(12):1439–49. 10.1097/TP.0b013e3181ff4ff3 21042237

[37] Wang GC Dash P McCullers JA Doherty PC Thomas PG. T cell receptor alphabeta diversity inversely correlates with pathogen-specific antibody levels in human cytomegalovirus infection. Sci Transl Med (2012) 4(128):128ra42. 10.1126/scitranslmed.3003647 PMC3593633 22491952

[38] Brochet X Lefranc MP Giudicelli V. IMGT/V-QUEST: the highly customized and integrated system for IG and TR standardized V-J and V-D-J sequence analysis. Nucleic Acids Res (2008) 36(Web Server issue):W503–8. 10.1093/nar/gkn316 PMC2447746 18503082

[39] Folch G Scaviner D Contet V Lefranc MP. Protein displays of the human T cell receptor alpha, beta, gamma and delta variable and joining regions. Exp Clin Immunogenet (2000) 17(4):205–15. 10.1159/000019140 11096259

[40] Szymczak AL Workman CJ Wang Y Vignali KM Dilioglou S Vanin EF. Correction of multi-gene deficiency in vivo using a single ‘self-cleaving’ 2A peptide-based retroviral vector. Nat Biotechnol (2004) 22(5):589–94. 10.1038/nbt957 15064769

[41] Nguyen TH Rowntree LC Pellicci DG Bird NL Handel A Kjer-Nielsen L. Recognition of distinct cross-reactive virus-specific CD8+ T cells reveals a unique TCR signature in a clinical setting. J Immunol (2014) 192(11):5039–49. 10.4049/jimmunol.1303147 24778446

[42] Purcell AW Ramarathinam SH Ternette N. Mass spectrometry–based identification of MHC-bound peptides for immunopeptidomics. Nat Protoc (2019) 14(6):1687–707. 10.1038/s41596-019-0133-y 31092913

[43] Koutsakos M Illing PT Nguyen THO Mifsud NA Crawford JC Rizzetto S. Human CD8+ T cell cross-reactivity across influenza A, B and C viruses. Nat Immunol (2019) 20(5):613–25. 10.1038/s41590-019-0320-6 30778243

[44] Escher C Reiter L MacLean B Ossola R Herzog F Chilton J. Using iRT, a normalized retention time for more targeted measurement of peptides. Proteomics (2012) 12(8):1111–21. 10.1002/pmic.201100463 PMC3918884 22577012

[45] Perez-Riverol Y Csordas A Bai J Bernal-Llinares M Hewapathirana S Kundu DJ. The PRIDE database and related tools and resources in 2019: improving support for quantification data. Nucleic Acids Res (2019) 47(D1):D442–d50. 10.1093/nar/gky1106 PMC6323896 30395289

[46] Krzywinski M Schein J Birol I Connors J Gascoyne R Horsman D. Circos: an information aesthetic for comparative genomics. Genome Res (2009) 19(9):1639–45. 10.1101/gr.092759.109 PMC2752132 19541911

[47] Yang C-WO Hung S-I Juo C-G Lin Y-P Fang W-H Lu IH. HLA-B*1502-bound peptides: implications for the pathogenesis of carbamazepine-induced Stevens-Johnson syndrome. J Allergy Clin Immunol (2007) 120(4):870–7. 10.1016/j.jaci.2007.06.017 17697703

[48] White KD Chung WH Hung SI Mallal S Phillips EJ. Evolving models of the immunopathogenesis of T cell-mediated drug allergy: The role of host, pathogens, and drug response. J Allergy Clin Immunol (2015) 136(2):219–34; quiz 35. 10.1016/j.jaci.2015.05.050 26254049 PMC4577472

[49] Wu Y Sanderson JP Farrell J Drummond NS Hanson A Bowkett E. Activation of T cells by carbamazepine and carbamazepine metabolites. J Allergy Clin Immunol (2006) 118(1):233–41. 10.1016/j.jaci.2006.03.005 16815161

[50] Mauri-Hellweg D Bettens F Mauri D Brander C Hunziker T Pichler WJ. Activation of drug-specific CD4+ and CD8+ T cells in individuals allergic to sulfonamides, phenytoin, and carbamazepine. J Immunol (1995) 155(1):462–72. 7602118

[51] Naisbitt DJ Britschgi M Wong G Farrell J Depta JPH Chadwick DW. Hypersensitivity reactions to carbamazepine: Characterization of the specificity, phenotype, and cytokine profile of drug-specific T cell clones. Mol Pharmacol (2003) 63(3):732–41. 10.1124/mol.63.3.732 12606784

[52] Naisbitt DJ Farrell J Wong G Depta JP Dodd CC Hopkins JE. Characterization of drug-specific T cells in lamotrigine hypersensitivity. J Allergy Clin Immunol (2003) 111(6):1393–403. 10.1067/mai.2003.1507 12789244

[53] Alvestad S Lydersen S Brodtkorb E. Cross-reactivity pattern of rash from current aromatic antiepileptic drugs. Epilepsy Res (2008) 80(2-3):194–200. 10.1016/j.eplepsyres.2008.04.003 18490142

[54] Hirsch LJ Arif H Nahm EA Buchsbaum R Resor SR Jr Bazil CW. Cross-sensitivity of skin rashes with antiepileptic drug use. Neurology (2008) 71(19):1527–34. 10.1212/01.wnl.0000334295.50403.4c 18981374

[55] Hyson C Sadler M. Cross sensitivity of skin rashes with antiepileptic drugs. Can J Neurol Sci (1997) 24(3):245–9. 10.1017/S0317167100021880 9276112

[56] Wang XQ Lang SY Shi XB Tian HJ Wang RF Yang F. Cross-reactivity of skin rashes with current antiepileptic drugs in Chinese population. Seizure (2010) 19(9):562–6. 10.1016/j.seizure.2010.09.003 20888266

[57] Seitz CS Pfeuffer P Raith P Brocker EB Trautmann A. Anticonvulsant hypersensitivity syndrome: cross-reactivity with tricyclic antidepressant agents. Ann Allergy Asthma Immunol (2006) 97(5):698–702. 10.1016/S1081-1206(10)61103-9 17165282

[58] Cardone M Garcia K Tilahun ME Boyd LF Gebreyohannes S Yano M. A transgenic mouse model for HLA-B*57:01-linked abacavir drug tolerance and reactivity. J Clin Invest (2018) 128(7):2819–32. 10.1172/JCI99321 PMC6025983 29782330

[59] Rowntree LC Nguyen THO Farenc C Halim H Hensen L Rossjohn J. A Shared TCR Bias toward an Immunogenic EBV Epitope Dominates in HLA-B*07:02-Expressing Individuals. J Immunol (2020) 205(6):1524–34. 10.4049/jimmunol.2000249 32817371

[60] Rowntree LC van den Heuvel H Sun J D’Orsogna LJ Nguyen THO Claas FHJ. Preferential HLA-B27 Allorecognition Displayed by Multiple Cross-Reactive Antiviral CD8(+) T Cell Receptors. Front Immunol (2020) 11:248. 10.3389/fimmu.2020.00248 32140156 PMC7042382

