# Supplementary figures and images for: Carbamazepine Induces Focused T Cell Responses in Resolved Stevens-Johnson Syndrome and Toxic Epidermal Necrolysis Cases But Does Not Perturb the Immunopeptidome for T Cell Recognition
Source: Front Immunol. 2021 Apr 12;12:653710. doi: 10.3389/fimmu.2021.653710 (PMC8071863; doi:10.3389/fimmu.2021.653710)

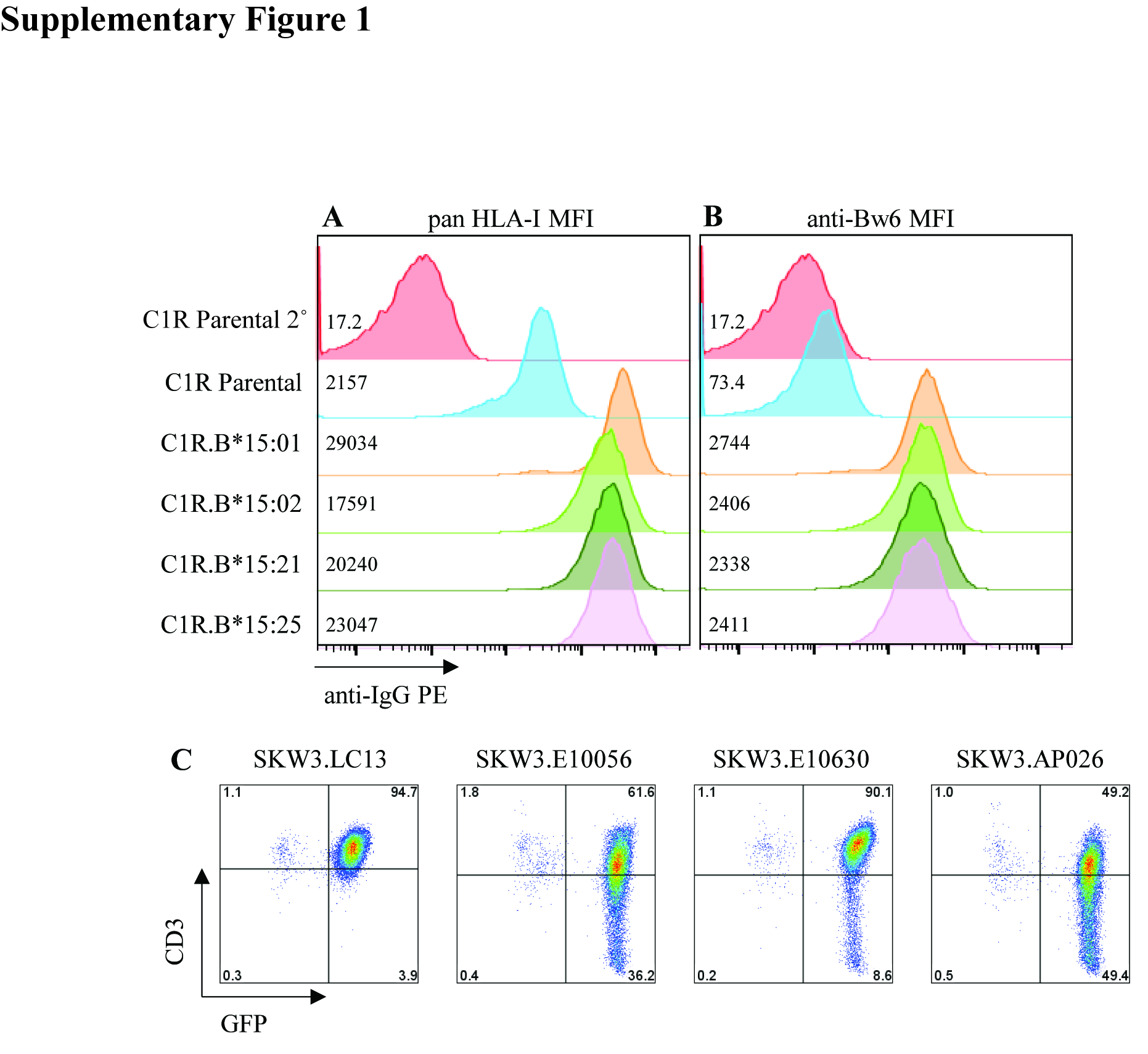

Supplement: Supplementary Figure 1 — HLA staining of Antigen Presenting Cells and SKW3.TCR expression. C1R Parental and transfected cells expressing different HLA-B15 allotypes were stained with primary antibodies (A) anti-human pan HLA-I (W6/32 hybridoma) and (B) anti-human HLA-Bw6 (HB152 hybridoma) followed by a goat anti-mouse IgG PE secondary antibody. A secondary (2°) antibody alone control was used for background staining. (C) All four SKW3 cell lines were periodically tested for TCR cell surface expression. A total of 30,000 cells were acquired on LSRII flow cytometer (BD) and data was analyzed using FlowJo software (version 10, BD). [file Image_1.tif]

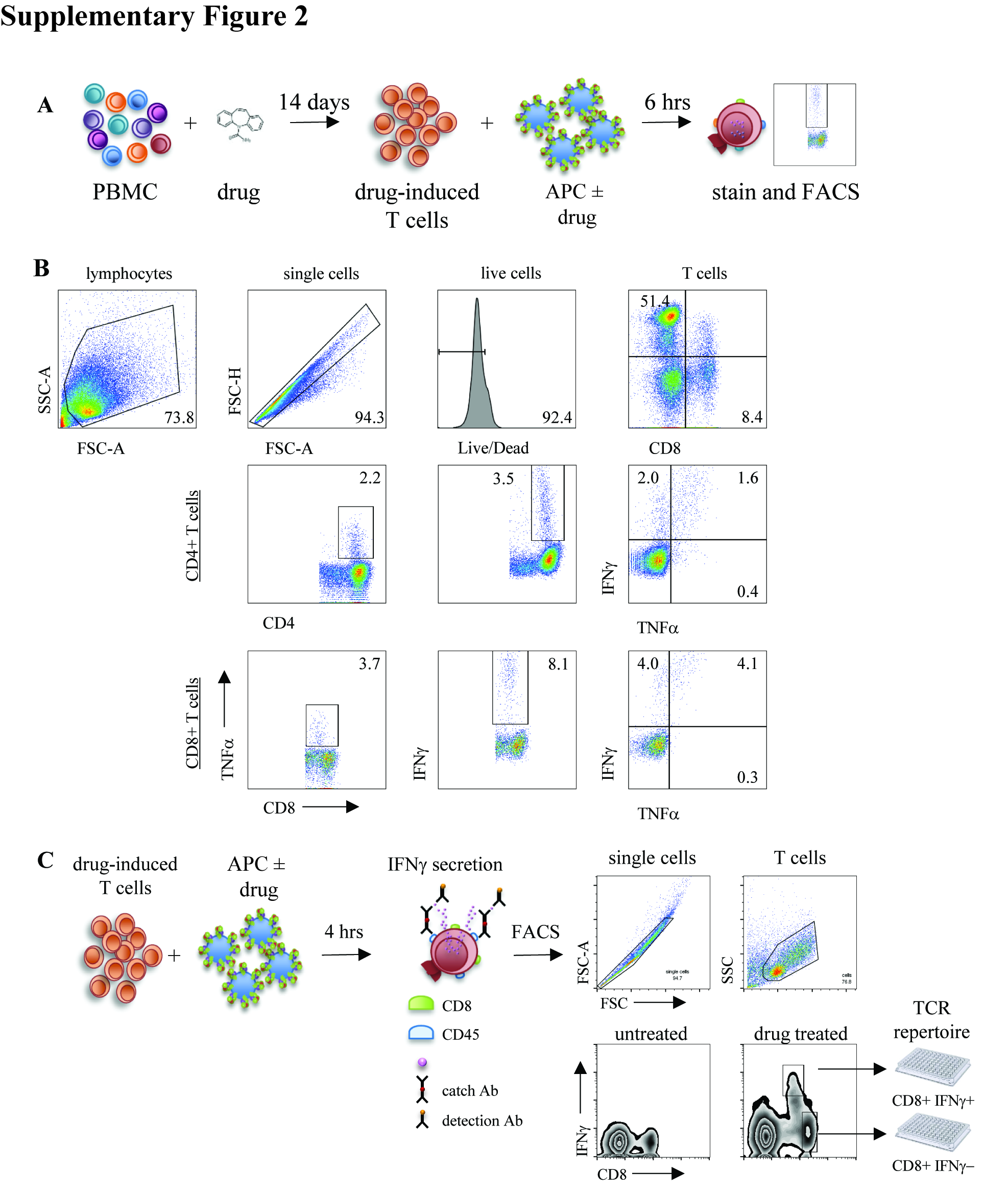

Supplement: Supplementary Figure 2 — In vitro expansion of CBZ-reactive T cells and cell sorting for αβTCR repertoire analysis. (A) Drug-reactive T cells were outgrown following 14 days of PBMC stimulation with 25 μg/mL CBZ. Day 14 T cells were restimulated for 6 hours with C1R cells expressing different HLA-B15 allotypes in the presence or absence of 25 μg/mL CBZ. T cell phenotype and activation was then measured by flow cytometry. (B) Gating strategy shown is representative data from SJS patient E10630. A maximum of 50,000 lymphocytes were acquired on LSRII flow cytometer (BD) and data was analyzed using FlowJo software (version 10, BD). (C) Day 14 CBZ-reactive T cells were restimulated with C1R.B*15:02 in either the absence (negative control) or presence of 25 μg/mL CBZ for 4 hours. Activated CD8+ T cells were detected using an IFNγ secretion assay. Single cell sorting of both CD8+ IFNγ- and CD8+ IFNγ+ T cell subsets for TCR repertoire analysis were performed using an Influx flow cytometer (BD). [file Image_2.tif]

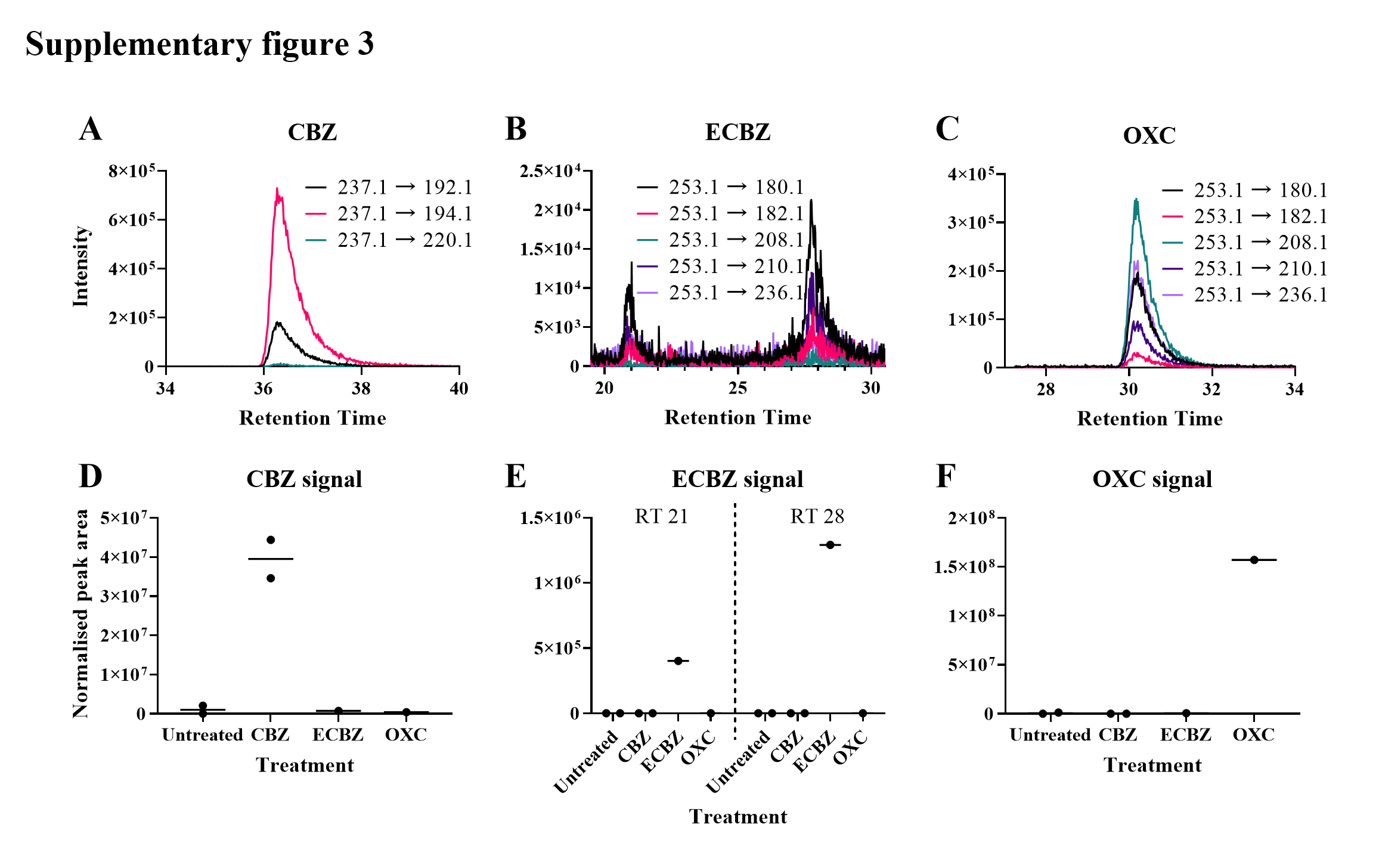

Supplement: Supplementary Figure 3 — Drug molecules co-purify with HLA-B*15:02. (A-C) Show raw intensities for transitions monitoring (A) CBZ in pool 10 of CBZ replicate 1, (B) ECBZ in pool 12 of ECBZ, (C) OXC in pool 13 of OXC. (D-F) summarize the normalized signal for CBZ (D), ECBZ (separated into the signal at 21 minutes (RT 21) and 28 minutes (RT 28) (E) and OXC (F) in pools 10, 12 and 13, respectively. [file Image_3.tif]

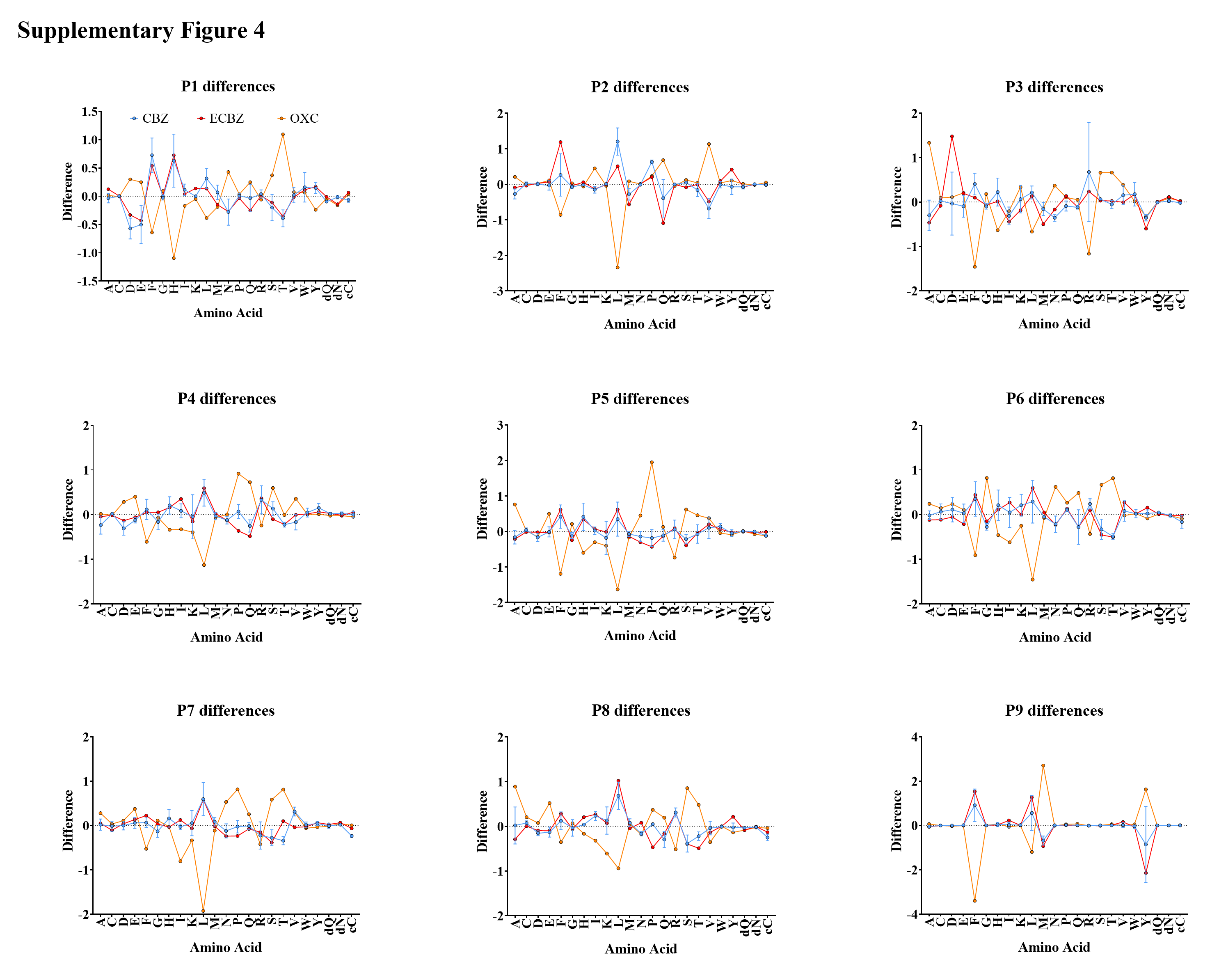

Supplement: Supplementary Figure 4 — Deviations in amino acid prevalence observed between treatments are minimal. Points represent differences between the amino acid prevalence at each position for drug/metabolite treatments as compared to the untreated control (mean of duplicate experiments). For CBZ, points are the difference of the mean of duplicate data sets with error bars showing standard error of the difference (calculated using Welch t-test). [file Image_4.tif]

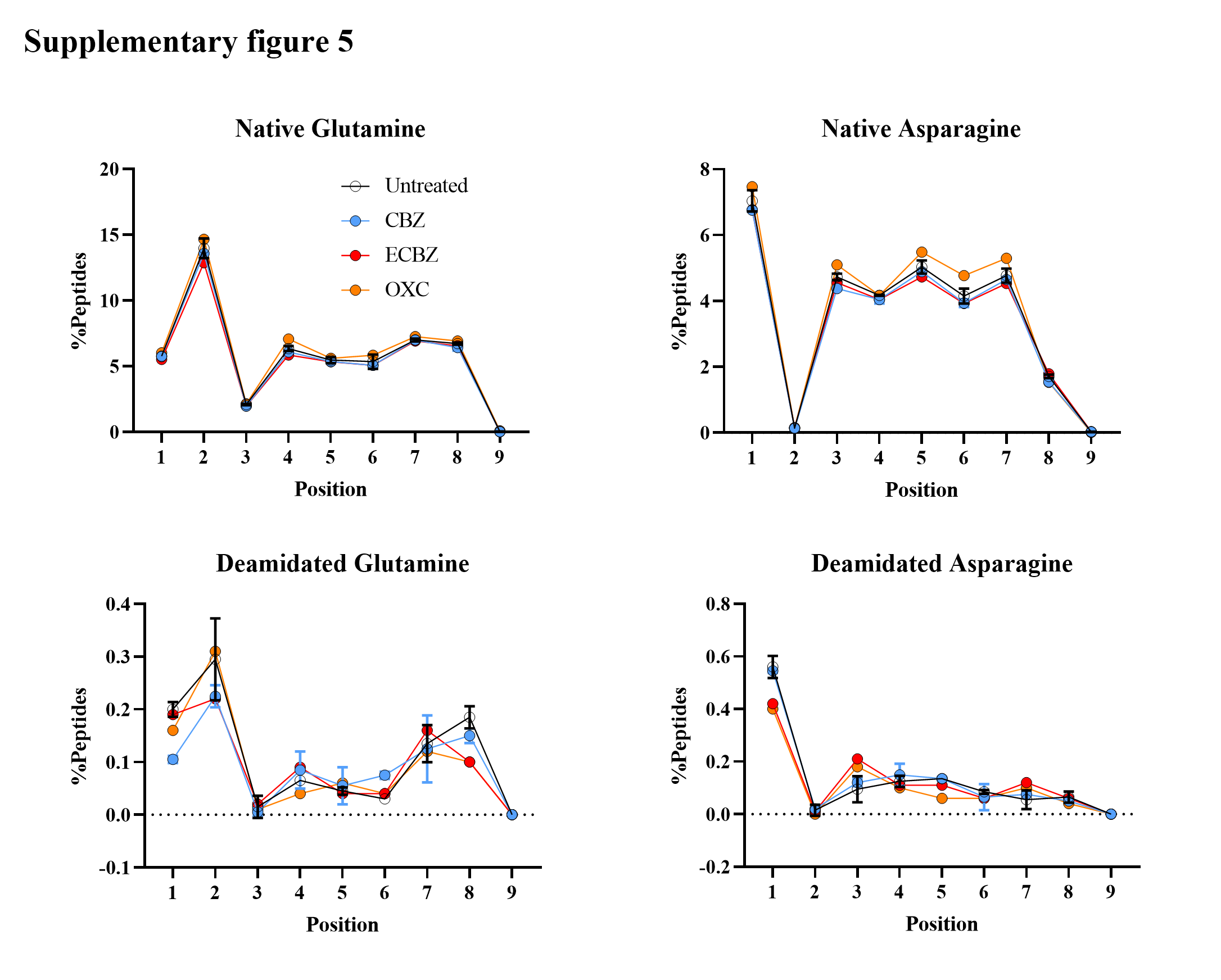

Supplement: Supplementary Figure 5 — No major alteration of the contribution of deamidated peptides to the HLA-B*15:02 immunopeptidome by drug treatment. Graphs show the percentage of 9mer peptides annotated as containing native Glutamine (top left), native Asparagine (top right), deamidated Glutamine (bottom left) and deamidated asparagine (bottom right) at each position of the peptide. For untreated and CBZ treatment, values are the mean of two replicate experiments, error bars show the standard deviation. ECBZ and OXC treatment are singlet values. [file Image_5.tif]

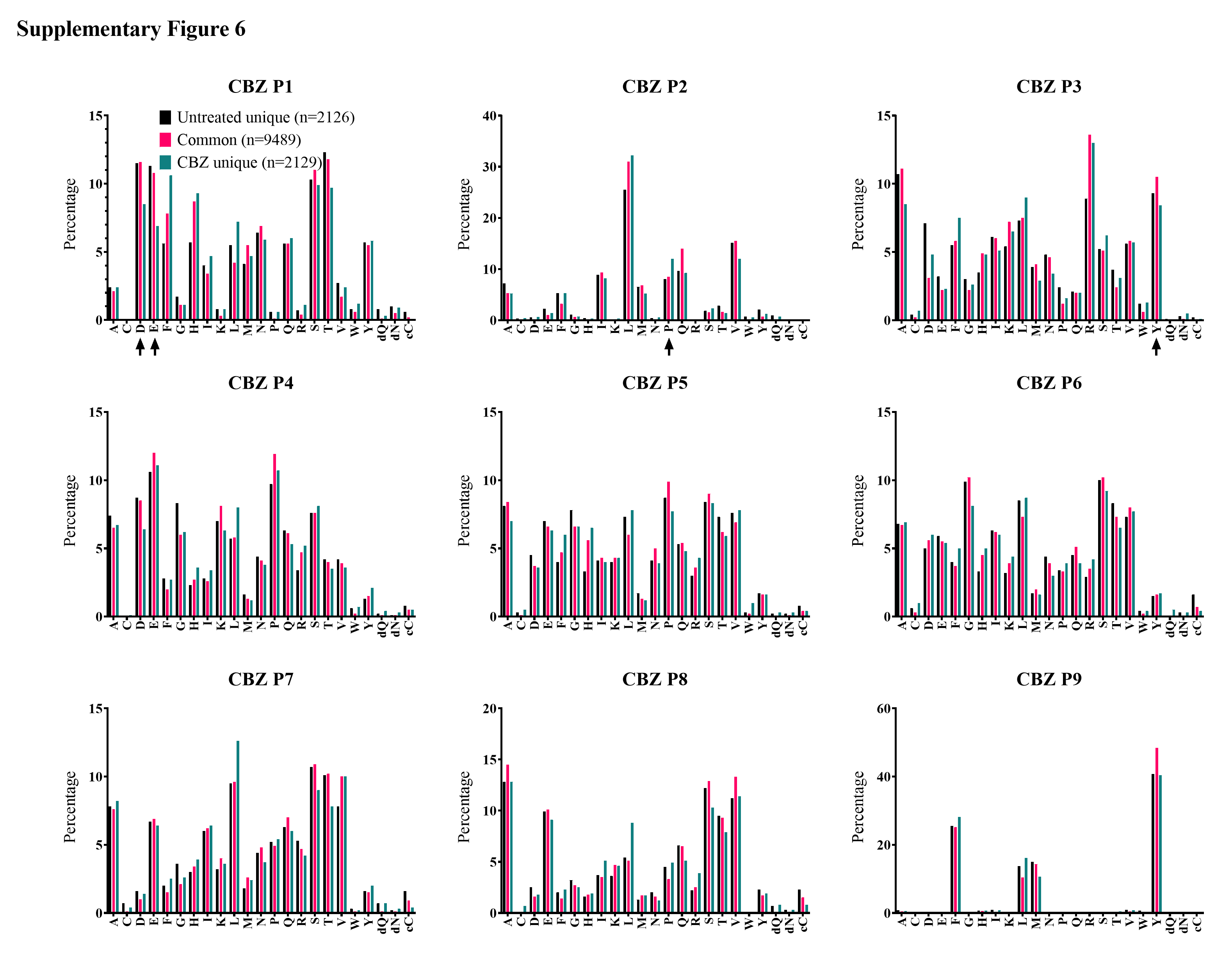

Supplement: Supplementary Figure 6 — Amino acid prevalence for 9mer peptides unique to CBZ treatment when compared to control conditions. Combined lists of peptides identified in at least 1 replicate of the control condition (untreated) and CBZ treatment were generated and compared to generate 3 categories; untreated unique – identified in at least one replicate of the untreated control, but not in either replicate of CBZ treatment, common – identified in at least one replicate of the untreated control and at least one replicate of the CBZ treatment, and CBZ unique – identified in neither replicate of the untreated control, but in at least one replicate of CBZ treatment. Bar graphs show the percentage of 9mer peptides possessing a given amino acid at each position of the peptide per category. n is the number of 9mer peptides in the category. Arrows indicate residues discussed in the text. [file Image_6.tif]

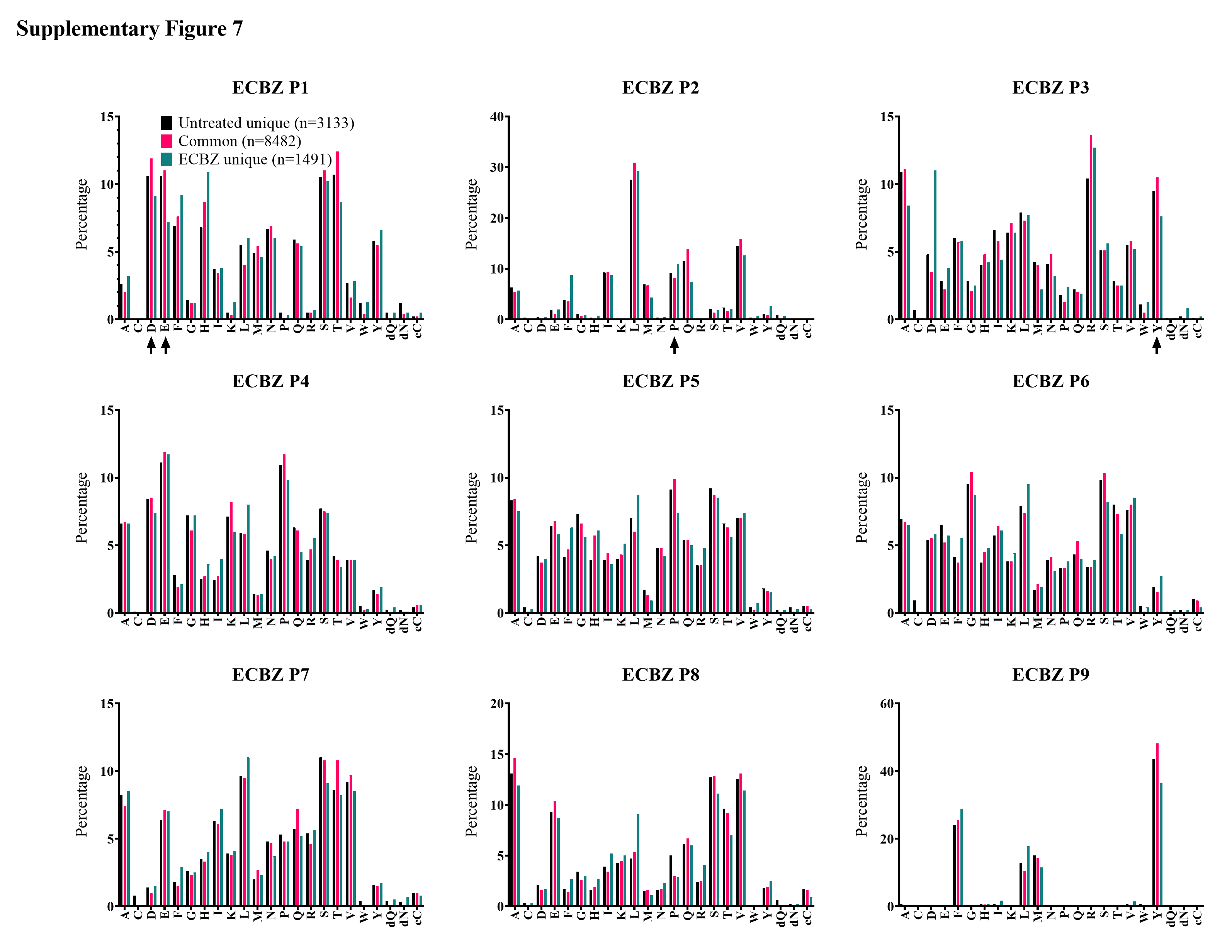

Supplement: Supplementary Figure 7 — Amino acid prevalence for 9mer peptides unique to ECBZ treatment when compared to control conditions. A combined list of peptides identified in at least 1 replicate of the control condition (untreated) was generated and compared to the ECBZ treatment data set to generate 3 categories; untreated unique – identified in at least one replicate of the untreated control, but not in ECBZ treatment, common – identified in at least one replicate of the untreated control and in the ECBZ treatment, and ECBZ unique – identified in neither replicate of the untreated control, but in ECBZ treatment. Bar graphs show the percentage of 9mer peptides possessing a given amino acid at each position of the peptide per category. n is the number of 9mer peptides in the category. Arrows indicate residues discussed in the text. [file Image_7.tif]

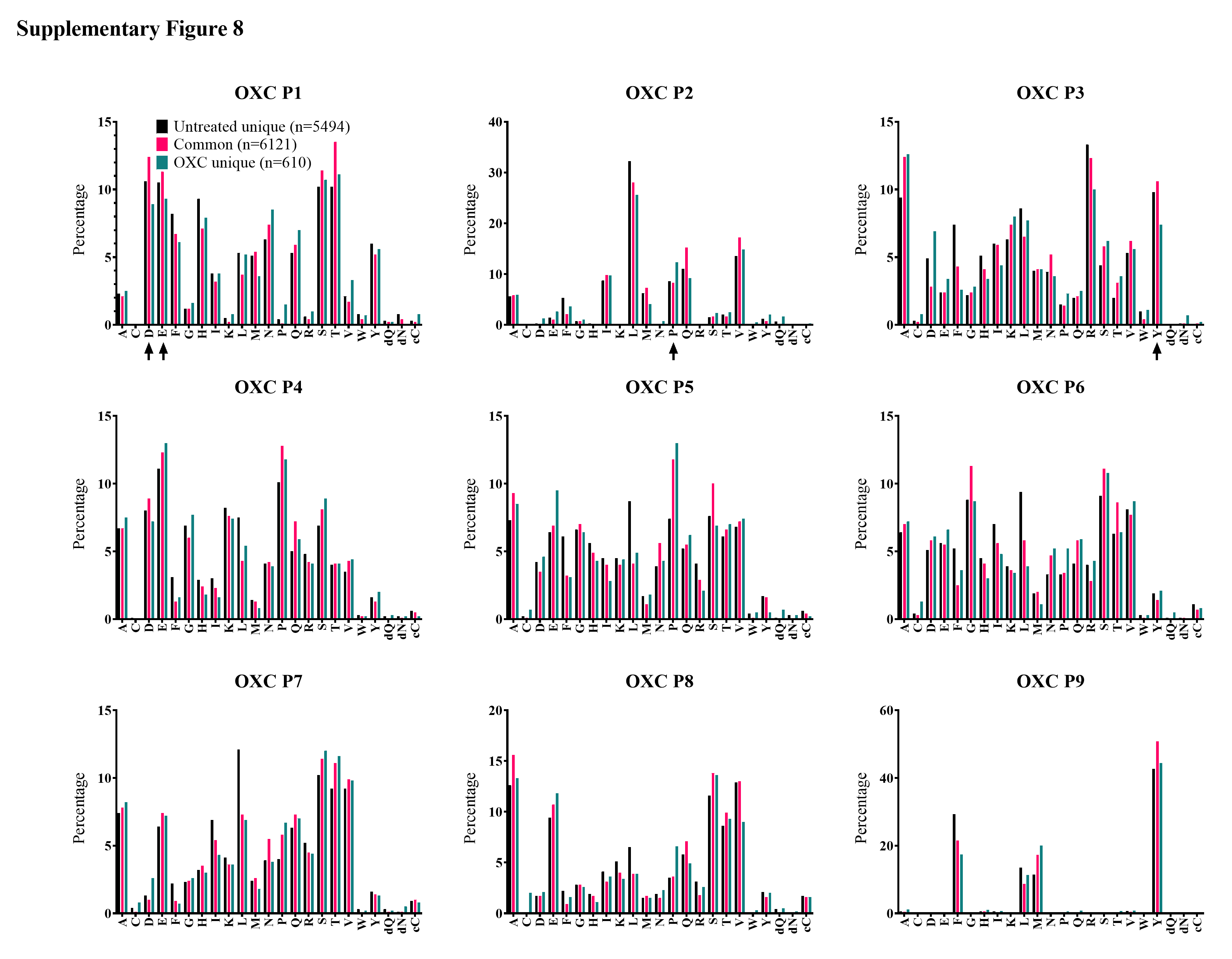

Supplement: Supplementary Figure 8 — Amino acid prevalence for 9mer peptides unique to OXC treatment when compared to control conditions. A combined list of peptides identified in at least 1 replicate of the control condition (untreated) was generated and compared to the OXC treatment data set to generate 3 categories; untreated unique – identified in at least one replicate of the untreated control, but not in OXC treatment, common – identified in at least one replicate of the untreated control and in the OXC treatment, and OXC unique – identified in neither replicate of the untreated control, but in OXC treatment. Bar graphs show the percentage of 9mer peptides possessing a given amino acid at each position of the peptide per category. n is the number of 9mer peptides in the category. Arrows indicate residues discussed in the text. [file Image_8.tif]
